# Endometriosis in the Mouse: Challenges and Progress Toward a ‘Best Fit’ Murine Model

**DOI:** 10.3389/fphys.2021.806574

**Published:** 2022-01-13

**Authors:** Katherine A. Burns, Amelia M. Pearson, Jessica L. Slack, Elaine D. Por, Alicia N. Scribner, Nazmin A. Eti, Richard O. Burney

**Affiliations:** ^1^Department of Environmental and Public Health Sciences, University of Cincinnati College of Medicine, Cincinnati, OH, United States; ^2^Department of Clinical Investigation, Madigan Army Medical Center, Tacoma, WA, United States; ^3^Department of Obstetrics and Gynecology, Madigan Army Medical Center, Tacoma, WA, United States

**Keywords:** endometriosis, mouse model, murine model, preclincal, lesions

## Abstract

Endometriosis is a prevalent gynecologic condition associated with pelvic pain and infertility characterized by the implantation and growth of endometrial tissue displaced into the pelvis via retrograde menstruation. The mouse is a molecularly well-annotated and cost-efficient species for modeling human disease in the therapeutic discovery pipeline. However, as a non-menstrual species with a closed tubo-ovarian junction, the mouse poses inherent challenges as a preclinical model for endometriosis research. Over the past three decades, numerous murine models of endometriosis have been described with varying degrees of fidelity in recapitulating the essential pathophysiologic features of the human disease. We conducted a search of the peer-reviewed literature to identify publications describing preclinical research using a murine model of endometriosis. Each model was reviewed according to a panel of ideal model parameters founded on the current understanding of endometriosis pathophysiology. Evaluated parameters included method of transplantation, cycle phase and type of tissue transplanted, recipient immune/ovarian status, iterative schedule of transplantation, and option for longitudinal lesion assessment. Though challenges remain, more recent models have incorporated innovative technical approaches such as *in vivo* fluorescence imaging and novel hormonal preparations to overcome the unique challenges posed by murine anatomy and physiology. These models offer significant advantages in lesion development and readout toward a high-fidelity mouse model for translational research in endometriosis.

## Introduction

Endometriosis is an estrogen growth-dependent, chronic inflammatory condition characterized by the implantation and growth of endometrial tissue outside the uterine cavity, most commonly on or within the pelvic peritoneum, ovary, and bowel (Burney and Giudice, 2012). A debilitating disease, endometriosis is strongly associated with pain and infertility, affecting 6–10% of adolescent girls and reproductive age women (Eskenazi and Warner, 1997) with potentially higher incidence from women going undiagnosed (Buck Louis et al., 2011). Persistent symptoms can significantly affect quality of life, with over half of affected women reporting a negative impact of the condition on their education, career, and relationships (De Graaff et al., 2013). The limited therapeutic options and frequent recurrence of disease symptoms present additional challenges. Patients with endometriosis evidence a higher utilization of outpatient and emergency room services, with the greatest economic burden occurring in the first year after diagnosis. The significant personal and societal impact of endometriosis highlight the importance of research efforts toward optimizing the approach to this complex disease (Giudice and Kao, 2004; Rogers et al., 2009; Simoens et al., 2012).

Although first described over 300 years ago, endometriosis remains a disease with significant knowledge gaps in both diagnosis and treatment (Nezhat et al., 2012). The persistence of these gaps is in part a reflection of a currently incomplete understanding of disease pathogenesis. The retrograde transit of viable endometrial tissue via open fallopian tube(s) into the peritoneal cavity during menstruation (Sampson, 1927) represents an evidence-based (Blumenkrantz et al., 1981; Halme et al., 1984) and widely accepted explanation for endometrial displacement. The discrepancy between the nearly universal prevalence of retrograde menstruation and the nearly 10% prevalence of endometriosis highlights the fundamental importance of endometrial implantation and growth in disease pathogenesis. Conceptually, several key steps including escape from immune clearance, attachment to peritoneal epithelium, invasion of the epithelium, establishment of neurovascularity and continued growth and survival are necessary for endometriosis to develop from retrogradely displaced endometrium (Lebovic et al., 2001; Burney and Giudice, 2012).

The absence of a non-surgical biomarker for the diagnosis and surveillance of endometriosis render longitudinal studies of women for the study of temporal relationships underpinning disease pathogenesis an unethical and impractical approach. Animal models of human disease are a potentially powerful resource for the study of pathogenesis, biomarker development and therapeutic discovery, particularly for complex progressive conditions such as endometriosis. The ultimate goal of any animal model is fidelity in the recapitulation of the human disease, and therefore the ideal species for endometriosis research is one that spontaneously develops the disease.

The female reproductive tract of non-human primates such as the baboon and rhesus macaque closely approximates that of humans in both form and function. Indeed, both species are reported to menstruate and spontaneously develop endometriosis (MacKenzie and Casey, 1975; D’Hooghe et al., 1996). To facilitate study, experimental induction of retrograde menstruation via surgical occlusion of the cervix resulted in the development of peritoneal endometriosis (D’Hooghe et al., 1994). Though ideal in terms of fidelity, the use of non-human primate species in endometriosis research is limited by the high cost of animal curation, length of time to develop endometriotic lesions, percentage of animals developing endometriosis, extended duration of gestation for fertility studies, and ethical considerations (D’Hooghe et al., 1994, 1996, 1997; D’Hooghe, 1997).

The mouse is widely embraced for preclinical model development in biomedical research due to several advantages. First, the smaller size, shorter estrous cycle and gestation and molecularly homogeneous background permit the study of larger groups of animals, thereby facilitating attainment of biological and statistical power in experimental research. Second, the mouse is a molecularly well-annotated species, allowing the researcher to leverage a large number of interrogative tools toward investigating complex multifactorial disease states. Finally, the ease of genetic manipulation and targeted alteration of candidate genes make the mouse particularly well suited for dissecting the molecular underpinnings of disease pathogenesis. Yet, the mouse, like other members of the rodent family, is generally not known to menstruate and does not develop endometriosis spontaneously. Consequently, previous studies modeling endometriosis using mice required induction of menstruation and transplantation of endometrium for the induction of endometriotic lesions. Interestingly, menstruation and spontaneous decidualization were reported in the spiny mouse (*Acomys cahirinus*), and its use was proposed as a more appropriate laboratory species for the modeling of menstrual disorders (Bellofiore et al., 2017); however, these mice also have a closed reproductive system and are quite fragile with challenging dietary preferences.

Since the first report of a mouse model of endometriosis in 1995, a large number of models have been described for disease induction (Cummings and Metcalf, 1995). A “best fit” murine model closely approximates key features of endometriosis, replicates molecular hallmarks, and minimizes interventions that may confound fidelity. Since the immune response and hormonal regulation play key roles in the pathogenesis of endometriosis, and the human reproductive organs (open reproductive system) are anatomically different than lower animals (closed reproductive system), several challenges exist to design a “best-fit” murine model to study the mechanisms related to initiation, development, and long-term response of endometriosis. Ideally, the model is genetically manipulable, immunocompetent, and hormonally intact. This review identifies key parameters for a “best-fit” model, summarizes published mouse models in the context of these parameters, and highlights the major challenges to generating a “best-fit” murine model for future preclinical studies.

## Parameters for a “Best-Fit” Murine Model of Endometriosis

### Spontaneous Endometrial Attachment and Growth in Lesion Development

Initially proposed by Sampson in the 1920’s, the retrograde menstruation theory of endometriosis is a widely accepted mechanism supported by multiple lines of epidemiologic, clinical, and experimental evidence (Sampson, 1927). A “best fit” model approximates this mechanism by introducing fresh, unbound endometrial fragments into the peritoneal cavity, thereby replicating the spontaneous implantation of displaced endometrium to ectopic sites. Thorough consideration of factors that influence lesion growth and development, such as hormones and genetic variance, is also fundamental to the selection of an ideal murine model. Dodds et al. (2017) used naturally cycling mice to investigate lesion development in two different strains, C57BL/6 and BALB/c. Spontaneous lesion development, when harvested 3 weeks post disease initiation, occurred at a significantly higher rate when induction occurs during proestrus than estrus, irrespective of strain (Dodds et al., 2017). C57/BL6 mice were more likely to develop dense-type lesions; BALB/c mice developed a more cystic lesion phenotype. This study suggests the cycle phase and strain-associated hormones and genetics can influence lesion pathogenesis. In contrast, Burns et al. (2012, 2018) demonstrated disease initiation (<72 h) in C57BL/6 mice is dependent on the immune system and independent of estrogen and ESR1; whereas, lesion growth is estrogen and ESR1 dependent (>72 h). These findings correspond to hormonal cycles in women – during menstruation, sex-steroid hormones are low and the immune system more active. Lesions are thought to grow when estrogen levels rise during the proliferative phase of the menstrual cycle (>5 days).

### Menstrual Phase Endometrium at Implantation

The transplantation of endometrium stripped of other uterine tissue, such as myometrium, is most consistent with the human disease process. Menstruation is an inflammatory process, characterized by an increase in a variety of tissue-resident and recruited immune cells with as high as 40% of menstrual fluid composed of neutrophils, macrophages, and uterine natural killer (uNK) cells (Salamonsen and Woolley, 1999; Salamonsen and Lathbury, 2000; Battersby et al., 2004; Maybin et al., 2011). Menstrual phase endometrium incorporates resident immune cells and inflammatory mediators typically found in human menstrual endometrium. Because mice do not spontaneously menstruate, donor females must be hormonally treated to induce menstrual endometrium for transplantation. Greaves et al. (2014) describe a model system using solely menstrual endometrium from a murine model of menstruation as the source of syngeneic mouse endometrial tissue. The importance of menstrual endometrium is further highlighted by studies performed in baboons (D’Hooghe et al., 1995). Endometriosis was more efficiently induced with menstrual versus luteal phase endometrium, as evidenced by the higher number and larger surface area of endometriotic lesions. Therefore, this parameter of a “best fit” model not only approximates human disease pathogenesis, but also enhances the efficacy of lesion development.

### Immunocompetence

Endometriosis is a chronic inflammatory disease, characterized by immune dysfunction and a marked increase in levels of pro-inflammatory cytokines in the peritoneal fluid (Halis and Arici, 2004). Though a complete discussion of endometriosis-associated inflammation and immune dysfunction is beyond the scope of this review, a role for inflammation as a hallmark feature of this disease is well established (Lebovic et al., 2001). Tumor necrosis factor alpha (TNFα) was observed to influence the establishment and progression of disease, whereas antagonists of TNFα were capable of inhibiting growth of established lesions in a mouse model (Altan et al., 2010). Likewise, in a preventive design, IL-12 (anti-inflammatory cytokine) administered to mice challenged with intraperitoneal endometrial material significantly reduced lesion development (Somigliana et al., 1999). Models using mice with an intact immune system are vital to understanding the role of immunologic and inflammatory pathways in pathogenesis studies as well as biomarker and therapeutic discovery. Important to note, several of the most used inbred strains are highly divergent in their immune response patterns due to mutations and polymorphisms. These inherent differences need to be considered when evaluating the immunologic and inflammatory pathways in generated disease. For example, FVB/NJ strains have germline deletions in the Tcrb-V8 gene, causing defects in natural killer T cell function, and potentially affecting their ability to produce TNFα. C57BL/6 mice are shown to have a TH1-type bias to pathogens, whereas BALB/c, A/J, and DBA/2 mice, tend toward a TH2-predominant response. Historically, inbred mouse strains are utilized as they provide genetic consistency and experimental reproducibility. However, recent studies suggest that inbred mice vary substantially from their outbred counterparts in their immune response to disease, suggesting inbred mice may not serve as an accurate model for the human condition. Thus, the of outbred mice in these models may more accurately reflect the role of immunologic and inflammatory pathways in the pathogenesis and progression of endometriosis.

### Intact Ovaries

Estrogen dependence on disease growth is an established pathophysiologic hallmark of endometriosis. In women, estradiol (E_2_) produced by the ovaries is the primary source of lesion support, and oophorectomy can be an effective therapeutic intervention to suppress symptoms. For an accurate model of the human condition, an intact hypothalamic-pituitary-ovarian axis in recipient females is preferable. Leaving the ovaries intact preserves physiologic estrogen production and allows for the evaluation of potential drug effects on normal hormonal cycling. Most importantly, maintaining the recipient ovarian microenvironment allows for the evaluation of endometriosis-associated infertility. Cohen et al. (2014) described the effect of induced endometriosis on oocyte and embryo quality in a murine model. Their data demonstrated that while the number of ovulated oocytes was not diminished, peritoneal endometriosis decreased oocyte quality and the number of embryos (fertilized oocytes) in mice with endometriosis. Further study of endometriosis in preclinical models with intact ovaries may help to delineate the pathophysiologic underpinnings of endometriosis-associated infertility (Meuleman et al., 2009).

### Longitudinal Assessment of Endometriotic Lesions

The natural progression and life cycle of endometriotic lesions represents a significant knowledge gap in the clinical approach to the disease. Clinically, the inability to reliably image peritoneal disease and the infeasibility of serial laparoscopies render this gap best approached using an animal model that facilitates the longitudinal *in vivo* assessment of disease burden. The use of luminescence represents an innovative approach that greatly enhances the longitudinal reach of preclinical endometriosis models (Defrere et al., 2009). Luminescence has allowed the resolution of endometriotic lesions that are difficult to detect macroscopically, and has the potential to afford a non-invasive evaluation of disease. Incorporation of luminescence is not only useful for diagnosis, but also for identification, visualization, and quantification of lesion burden. Hirata et al. (2005) originally created a green fluorescent protein (GFP) mouse model of endometriosis in 2005 to more easily locate lesions in the peritoneal cavity. Wieser et al. (2012) then used GFP transgenic donor mice to increase sensitivity in the identification of endometriotic lesions and to more accurately quantify lesion size and growth rates in response to an all-*trans*-retinoic acid treatment. Endometriotic tissue originating from donor endometrium can be clearly delineated from surrounding recipient tissues with the use of luminescence and/or immunohistochemistry. Wilkosz et al. (2011) successfully monitored cellular exchange between host peritoneum and endometrial tissue with the use of allogeneic transplants from transgenic GFP positive donor mice to wild-type recipients. Use of GFP-expressing tissue provided strong evidence for both ingrowth and outgrowth of blood vessels during the development of an endometriotic lesion.

Newer transgenic strains are commercially available that constitutively express fluorophores at emission spectra outside the autofluorescent range, thereby optimizing the signal to noise ratio at detection. The use of donor endometrium expressing red fluorescent protein (RFP) in disease induction facilitated non-invasive monitoring of lesion growth and regression for up to 33 days post transplantation in wild-type recipient mice (Wang et al., 2014). Ferrero et al. (2017) developed a homologous murine model utilizing the fluorescent reporter mCherry, which resulted in deeper penetration and a more robust signal-to-noise ratio to longitudinally monitor lesions *in vivo*. In addition to luminescence, other methods of non-invasive *in vivo* analysis are described for visualization of the development of endometriotic lesions in mice. Körbel et al. (2010) successfully utilized high-resolution ultrasound imaging for repeat *in vivo* analysis of lesion development in their murine model. Ultrasound afforded quantitative determination of overall lesion volume and stromal tissue and cyst content. Magnetic resonance imaging (MRI) for *in vivo* volumetric measurement of endometrial implants has also been attempted (Silveira et al., 2013). The use of luminescence and high-resolution imaging modalities have made non-invasive longitudinal assessment possible in murine models of disease.

### Iterative Endometrial Transplantation

In a study of laparoscopies timed to menstruation, Halme et al. (1984) revealed retrograde menstruation to be a nearly universal phenomenon in women with patent oviducts (Halme et al., 1984). Endometriosis is a dynamic disease characterized by the establishment of new lesions each cycle and the continued progression of established lesions. The repetitive introduction of endometrium into the peritoneal cavity is therefore an important model parameter toward recapitulating the human condition.

## Evaluation of Published Mouse Models in the Context of “Best-Fit” Murine Model Parameters

### Methods of Induction

Chief among the limitations in establishing a murine system to model endometriosis are the fundamental differences between mouse and human reproductive physiology and anatomy. In particular, the murine tubo-ovarian junction is contained within a bursa that precludes retrograde flow from the uterus into the peritoneum. Consequently, the disease must be induced in mice. This step was accomplished by surgically grafting fragments throughout the peritoneal cavity or by injecting endometrium into the peritoneal cavity.

#### Surgical Engraftment

The first reported murine model of endometriosis induced lesions by surgical engraftment. This model involves the placement, by suture or adhesion, of uterine tissue into the peritoneal cavity of the same or a recipient mouse (Table 1). The advantages of this model allow for ease of lesion location and uniform development of lesions from the same starting size. However, from engraftment locations to the amount (i.e., size and number) of uterine material given, methods of engraftment vary between studies.

**TABLE 1:** Murine models of endometriosis: surgical engraftment method.

Strain	Induction age	D:R	Induction tissue	Donor primed	Rec. ovex’ed	E2	Tissue amount given	Stripped EM	References
D: ubiquitin-GFP R: C57BL/6	6 wk	1:2	UHs	N, in diestrus	N	N	3 mm fragments	N	Chen et al., 2021
C57BL/6J	12–18 wk	1:1	Minced UH	N	N	N	Three 2 mm^2^ pieces	N	Santorelli et al., 2021
C57BL/6	4 wk	NM	UHs	NM	N	Y	Four 2 mm punches	N	Sharma et al., 2021
NU/NU Nude mouse (Crl:NU-Foxn1*^nu^*)	5 wk	NA	HS	NA	Y	Y	Two to three 3–5 mm^2^ strips	NA	Tejada et al., 2021
C57BL/6N	8–10 wk	1:1	UHs	NM	NM	NM	Two 3 mm^2^ punches	N	Symons et al., 2020
C57BL/6N	3 mo	NM	UH	N, in diestrus	N	N	0.8–1 mm pieces	N	Mishra et al., 2020
C57BL/6	8–10 wk	Auto	UH	E2 capsule	N	N	One UH	N	Li et al., 2020
BALB/c	6 wk	1:1	UH	NM	Y	Y	Two 2 mm pieces	N	Chang et al., 2020
C57BL/6 WT Flt1 TK^–/–^	8 wk	1:1	UHs	NM	Y	Y	Two 3 mm punches	N	Hattori et al., 2020
C57BL/6	8 wk	1:1	UH pellet	NM	N	N	One uterus	N	Hayashi et al., 2020
C57BL/6J, AI6(RCL-ZsGreen)	7–8 wk	NM	UH	N, in diestrus	N	N	Four 3 mm^3^ pieces	N	Tal et al., 2019
C57BL/6, CBA/J, BALB/c	6 wk	1:2	UH	NM	N	Y	One 5 mm piece	N	Peyneau et al., 2019
WT, Gal-3-deficient	8 wk	NM	UH	NM	N	N	One UH	N	Mattos et al., 2019
Soft Swiss Nude Mice	6–8 wk	NA	HS	NA	Y	Y	One implant	NA	Martinez et al., 2019
C57BL/6	10 wk	Auto	UH	NA	N	Y	3 mm fragment	N	Chadchan et al., 2019
D: GFP + TK–/–TG R: Flt TK–/–	8 wk	NM	UHs	E2	Y	Y	Four 3 mm punches	N	Sekiguchi et al., 2019
B6CBA/F1	D: 9 wk, R: 5 wk	1:1	UH	NM	N	N	Two 2 mm pieces	N	Cohen et al., 2015
Cccn-1 null mice	NM	NM	UHs	N, in diestrus	Y	Y	Two 3 mm punches	N	Zhao et al., 2014
CD-1	8 wk	NM	UHs	NM	N	N	Two UH	N	Naqvi et al., 2014
D: GFP R: WT	8 wk	1:1	Minced UH	N, in estrus	N	N	3 mm^2^ pieces	N	Machado et al., 2014
Outbred ICR	6 wk	Auto	UHs	E2	Y	Y	NM	N	Liao et al., 2014
C57BL/6J	8 wk	1:2	UHs	N, cages synchronized	N	N	Six 2 mm punches	N	Kumar et al., 2014
C57BL/6	D: 9 wk, R: 5 wk	1:1	UHs	NM	N	N	Two 2 mm punches	N	Cohen et al., 2014
B6C3F1-auto R: CD-1	5–6 wk	Auto	UH/HS	NA	CD1’s	Y	Five 1-2 mm pieces	N	Silveira et al., 2013
C57BL/6 eGFP	10–12 wk	NM	UHs	NM	Y/N	Y	Two 2 mm pieces	N	Wilkosz et al., 2011
BALB/c	2 mo	Auto	UHs	NA	N	N	Three 4 mm^2^ pieces	N	Ricci et al., 2011
CD-1	8 wk	1:2	UHs	NM	N	N	Two pieces	N	Kulak et al., 2011
129 × 1/SvJ 129S6/SvEvTec C57BL/6 GFP	8 wk	NM	UHs	N	N	N	Four/Six 2 mm punches	N	Becker et al., 2011
C57BL/6	8–9 wk	auto	UH	N	N	Y	Four pieces	N	Lu et al., 2010
ddY	8–11 wk	auto	UH	NA	N	N	Two 3 mm^3^ pieces	N	Kusakabe et al., 2010
C57BL/6	12–16 wk	1:1/auto	UH	N, in estrus	N	N	Two 2-3 mm^2^ punches	N	Körbel et al., 2010
BALB/c	2 mo	NM	UH	NM	N	N	Three 4 mm^2^ pieces	N	Bilotas et al., 2010
ICR	8 wk	1:1	UH	NM	NM	NM	Two 5 mm^2^ pieces	N	Umezawa et al., 2009
CD-1	8 wk	1:2	UH	NM	N	N	Two pieces	N	Lee et al., 2009
C57BL/6	6–8 wk	Auto	UHs	N	N	N	Four 2 mm punches	N	Fainaru et al., 2008
C57BL/6	8–9 wk	1:2	UH	E2	N	N	Three pieces	N	Matsuzaki et al., 2008
D: GFP R: C57BL/6	8 wk	1:1/auto	UHs	NM	Y	Y	Seven 2 mm punches	N	Becker et al., 2008
C57BL/6	8 wk	Auto	UH	N, in estrus	N	Y	Seven 2 mm punches	N	Efstathiou et al., 2005
B6C3F1 mice	60 d	Auto	UH	NA	Y	Y	Three 2.5–3 mm pieces	N	Cummings and Metcalf, 1995

*Ovex, ovariectomy; EM, endometrium; E2, estrogen; D, donor; R, recipient; auto, autotransplantation of self-tissue; UH, uterine horn; UHs, two uterine horns; DT, decidualized tissue; HS, human sample; N, no; Y, yes; NM, not mentioned; NA, not applicable; h, hours; d, day; wk, week; mo, month; BNF, beta-naphthoflavone.*

Models for endometriosis lesion development are typically autologous, syngeneic, or heterologous. In heterologous engraftment models, human samples are either implanted, sewn into the peritoneal cavity or intestine, or adhered to the peritoneal wall of immune incompetent murine recipients (e.g., the Soft Swiss Nude Mouse (Martinez et al., 2019) or the NU/NU Nude Mouse [Crl:NU-Foxn1*^nu^*]) (Tejada et al., 2021). Autologous and syngeneic models utilize murine tissue donors. Tissue samples have been sutured with 4-0 nylon suture (Cummings and Metcalf, 1995), 7-0 polypropylene suture (Cohen et al., 2014), silk suture (Fainaru et al., 2008), braided silk suture (Efstathiou et al., 2005; Becker et al., 2008), or stuck to the wall with adhesive such as bonding agent 3M vetbond (Symons et al., 2020). Uterine tissue engrafted varied from one large (5 mm) piece (Peyneau et al., 2019) to up to six (2 mm) tissue punches (Kumar et al., 2014). Tissue was sutured or adhered to the peritoneum (Wilkosz et al., 2011; Mattos et al., 2019; Tal et al., 2019; Chang et al., 2020; Hattori et al., 2020; Li et al., 2020; Symons et al., 2020; Yoshino et al., 2020; Chen et al., 2021; Santorelli et al., 2021), intestinal mesentery (Mishra et al., 2020), abdominal wall (Peyneau et al., 2019), bowel mesentery (Ricci et al., 2011), and ovaries (Hayashi et al., 2020). Lesions sutured or adhered in the peritoneal cavity display characteristics similar to human lesions. Lesions tend to be cyst-like and fluid filled (Zhao et al., 2014; Cohen et al., 2015), with some adhesions (Kumar et al., 2014), and characterized by the presence of glands and stroma throughout (Mattos et al., 2019). Lesions varied in color from white, yellow, red, to chocolate brown (Yoshino et al., 2006; Fainaru et al., 2008).

In the engraftment-based model, lesions are easy to locate and lesion number does not vary among animals. Sanchez et al. proposed this model may best mimic mature endometriosis (Sanchez et al., 2017). However, in other contexts, this model has several recognized limitations. First, surgically implanted lesions bypass the attachment phase of the disease, limiting the utility of this type of model in the study of early disease pathogenesis and prevention. Specifically, this approach does not account for the interaction between the peritoneum and ectopic endometrial cells in the early development and growth of endometriosis, and this model does not allow for the study of spontaneous lesion attachment and varying location development. Second, surgically engrafted uterine biopsies often include non-endometrial tissues such as myometrium and serosa, which is inconsistent with the histology of retrograde menstruum in the human condition. None of the engraftment studies reviewed here removed the myometrium from the uterine tissue samples before implantation, although several suture the uterine tissue to the peritoneal wall with the endometrial side facing the peritoneal cavity (Santorelli et al., 2021) or intestinal serosa (Ricci et al., 2011). Finally, suture material and/or technique and healing process associated with surgical engraftment may alter the typical sequence of lesion development and may confound model readout, particularly angiogenesis, which suture material is known to alter (Wilkosz et al., 2011; Wang et al., 2013).

#### Intraperitoneal Injection

In contrast to surgical engraftment, injection-based models generate endometriotic lesions by intraperitoneal challenge with endometrial tissue from a syngeneic donor (Table 2). First described by Somigliana et al. (1999), recipient mice develop lesions on the peritoneum, perivesical adipose tissue, the intestinal surface, and/or the uterine surface. This model allows for the study of the initial stages of the disease which include angiogenesis, defective apoptosis, endometrial proliferation, and inflammation (Sanchez et al., 2017). The most frequently reported location of lesions in this model is the ventral abdominal wall. Importantly, the less invasive nature of injection relative to surgical engraftment facilitates iterative seeding of endometrial fragments as a recapitulation of monthly retrograde menstruation in the human condition. Since lesions are not sutured, endometrial-mesothelial interactions and angiogenesis may be studied more reliably than with the engraftment model.

**TABLE 2:** Murine models of endometriosis: intraperitoneal injection method.

Strain	Induction age	D:R	Induction tissue	Donor primed	Rec. ovex’ed	E2	Tissue amount given	Menstrual EM	Stripped EM	Suspended uterine material	References
D: CAG-luc-GFP R: WT FVB	8–12 wk	1:1	DT	E2 then P4	Y/N	Y/N	40 mg	Y	Y/N	Saline	Dorning et al., 2021
D: WT R: CD206-DTR	12–20 wk	1:2	Minced UH	E2	N	N	NM	N	NM	PBS	Ono et al., 2021
D: ICR R: C57BL/6	8 wk	1:1	DT	E2 and P4	Y	Y	One UH	Y, w/oil	N	Saline	Kim et al., 2020
C57BL/6	8 wk	1:2	Uterine fragments	E2	N	N	One UH	N	N	HBSS	Fattori et al., 2020
BALB/c	5 wk	1:1	UH	NM	NM	Y	One 1 cm piece	N	N	PBS	Woo et al., 2020
BALB/c	6 wk	1:2 MD	Uterine fragments	E2	N	N	One UH	N	N	Saline	Yan et al., 2019
D: GFP C57BL/6 R: C57BL/6	8 wk	1:1	DT	E2 then P4	N	N	40 mg	Y, w/oil	Y	PBS	Horne et al., 2019
D: GFP C57BL/6 R: C57BL/6	8 wk	1:1	DT	E2 then P4	Y	N	40 mg	Y, w/oil	N	PBS	Forster et al., 2019
C57BL/6	NM	2:1 MD	UH	E2	N	N	Fifteen 1 mm pieces	N	Y	PBS	Yuan et al., 2018
BALB/c, C57BL/6 (GFP)	8 wk	1:2	Minced UH	E2	N	N	One UH	N	Y	Saline	Sanchez et al., 2017
C57BL/6, BALB/c	9–15 wk	1:1	UH	NM	N	N	7.5–40 mg varied	N	Y	Saline	Dodds et al., 2017
C57BL/6, BALB/c	5 wk	2:1	Minced UH	E2	N	N	NM	N	Y/N	Saline	Ruiz et al., 2016
D: Klf9 R: C57BL/6J, Klf9, WT	9–10 wk	NM	Minced UH	NM	N	N	40 μg	N	Y	PBS	Heard et al., 2016
BALB/c	6 wk	1:2	Minced UH	NM	Y	Y	50 mg	N	N	Saline	Uegaki et al., 2015
MacGreen WT	8 wk	1:1	DT	E2	Y	Y	40 mg	Y	Y	PBS	Greaves et al., 2014
D: GFP R: C57BL/6	NM	NM	Minced UH	E2	NM	Y	∼35 mg	N	N	PBS	Pierzchalski et al., 2014
Fat-1 mice, WT, 12/15-LOX-KO	6–8 wk	1:2	Minced UH	E2	Y	Y	NM	N	Y	PBS	Tomio et al., 2013
BALB/c	6 wk	1:2	Minced UH	E2	Y	Y	1/2 uterus	N	N	Saline	Takai et al., 2013
D: GFP R: C57BL/6	NM	NM	Minced UH	E2	NM	Y	35 mg	N	NM	PBS	Wieser et al., 2012
BALB/c	6 wk	1:2	Minced UH	E2	Y	Y	46 ± 5 mg/mouse	N	Y	Saline	Itoh et al., 2011
BALB/c	6–8 wk	1:2	Minced UH	E2	Y	Y	NM	N	N	Saline	Pittaluga et al., 2010
FVB, C57BL/6, CSF-1, op/op	6–8 wk	1:2	UH	E2	N	Y	35 mg	N	Y	PBS	Jensen et al., 2010
C57BL/6	NM	1:1	Minced UH	PMSG	N	N	One uterus	N	N	PBS	Altan et al., 2010
BALB/c	8 wk	1:2	Minced UH	NM	Y	Y	One UH	N	Y	PBS	Chen et al., 2009
BALB/c	8 wk	1:2	Minced UH	E2	N	N	Pieces < 1 mm	N	N	PBS	Bacci et al., 2009
D: eGFP R: C57BL/6	6–8 wk	NM	Minced UH	N, taken in estrous	NM	N	40 mg	N	Y	DMEM	Nowak et al., 2008
BALB/c	6–8 wk	1:2	Minced UH	E2	Y	Y	1/2 uterus	N	Y	PBS	Yoshino et al., 2006
D: GFP R: C57BL/6	6–8 wk	1:2	Minced UH	E2	Y	Y	One UH	N	Y	PBS	Hirata et al., 2005
Swiss Webster	8–10 wk	1-2:1	Minced UH	N	N	NM	1–2 × 10^5^ cells	NA	NA	PBS	Cao et al., 2004
C57BL/6 BALB/c	6–8 wk	1:2	Minced UH	E2	Y	Y	15 mg	N	Y	Saline	Somigliana et al., 1999

*Ovex, ovariectomy; EM, endometrium; E2, estrogen; P4, progesterone; D, donor; R, recipient; MD, mixed donors; UH, uterine horn; DT, decidualized tissue; N, no; Y, yes; NM, not mentioned; NA, not applicable; h, hours; d, days; wk, week; PMSG, pregnant mare serum gonadotrophin; PBS, phosphate buffered saline.*

For intraperitoneal injection models using decidualized endometrium, decidualization is often induced by injecting oil (Greaves et al., 2014; Forster et al., 2019; Horne et al., 2019; Kim et al., 2020) into, or by scratching (Ferrero et al., 2017), the uterine horn and harvesting the decidualized tissue with forceps. A variety of hormonal paradigms in ovariectomized or intact female mice are described to induce endometrial decidualization prior to harvest (Greaves et al., 2014; Ferrero et al., 2017; Forster et al., 2019; Horne et al., 2019; Kim et al., 2020). The uterine horn is opened longitudinally and the decidualized endometrium is dissected or scraped from the underlying myometrium (Kim et al., 2020). Additionally, decidualization can be induced by scratching the antimesometrial lumen with a 27-G needle inside the uterine horn (Ferrero et al., 2017). Models that utilize minced uterine tissue taken from full-thickness uterine horns of a donor mouse, strip away the fat and muscle, and may or may not strip off the myometrium before injecting the minced tissue into the peritoneal cavity (Sanchez et al., 2017). Harvested tissue is suspended in 200 – 500 μl of solution before injection into the peritoneal cavity. Solutions vary between studies, and include saline (Pittaluga et al., 2010; Uegaki et al., 2015; Ruiz et al., 2016; Dodds et al., 2017; Sanchez et al., 2017; Yan et al., 2019; Kim et al., 2020), Hank’s Balanced Salt Solution (HBSS) (Fattori et al., 2020), phosphate-buffered saline (PBS) (Bacci et al., 2009; Chen et al., 2009; Altan et al., 2010; Jensen et al., 2010; Wieser et al., 2012; Tomio et al., 2013; Greaves et al., 2014; Heard et al., 2016; Yuan et al., 2018; Horne et al., 2019; Woo et al., 2020), and warmed Dulbecco’s Modified Eagle Medium (DMEM) (Nowak et al., 2008). The solutions chosen mimic the salinity and pH of the peritoneal cavity and maintain viability of the uterine tissue.

Though intraperitoneal injection of endometrium more closely approximates the pathophysiology of retrograde menstruation than surgical implantation methods, there is more variability in the number, distribution, and phenotype of lesions associated with the injection method. Lesions in this model are often located in the fatty tissue around the bladder (Yuan et al., 2018), the parietal peritoneum, and the visceral peritoneum of the uterus and intestines (Greaves et al., 2014). On average, this model produces 2–3 true lesions that contain epithelial lined glands with organized stroma, immune infiltration of hemosiderin laden macrophages, and fibrotic areas (Hsu et al., 2010). Cystic lesions are fluid-filled nodules ranging in color from white to pink to tan featuring endometrial glands and stroma. Cystic lesions are vascularized and infiltrated with inflammatory cells (Somigliana et al., 1999; Hirata et al., 2005; Uegaki et al., 2015; Dodds et al., 2017). Dense lesions were black/brown and dark red in color, filled with hemosiderin macrophages and other immune cells, and encapsulated by connective tissue. The lesions are of variable size and weight which may be due to the variable amount of tissue and fragment sizes injected among different groups. A key variable among groups was the needle size used for injection; needle sizes varied by group, ranging from 18-gauge needles up to 27-gauge needles [e.g., 18-gauge needle (Hirata et al., 2005; Yoshino et al., 2006; Chen et al., 2009; Altan et al., 2010; Jensen et al., 2010; Wieser et al., 2012; Tomio et al., 2013; Fattori et al., 2020; Ono et al., 2021), 19-gauge needle (Greaves et al., 2014), 20-gauge needle (Nowak et al., 2008), 21-gauge needle (Dodds et al., 2017), 25-gauge needle (Yuan et al., 2018), and 27-gauge needle (Heard et al., 2016)]. Optimization of injection methods to achieve greater reproducibility in terms of number and distribution of lesions is needed. Due to the difficulty in reliably locating lesions, particularly for longitudinal analyses, a variety of luminescence strategies (e.g., GFP uterine tissue) have evolved.

Dorning et al. (2021) compared four variants of the intraperitoneal injection method: decidualized tissue into an ovariectomized, but E2 supplemented, recipient (DO), decidualized tissue into a hormonally intact recipient (DI), minced naïve endometrium from cycling mice into hormonally intact recipients (NI), and full thickness uterine fragments, including the myometrium, from cycling mice into hormonally intact recipients (MI). Lesion progression was longitudinally analyzed at 7, 21, and 42 days by *in vivo* imaging of luciferase bioluminescence. At 7 days, 90% of DO and DI, 96.6% of NI, and 100% of MI mice evidenced lesions. At 42 days, bioluminescent lesions were observed in 40% DO, 50% DI, 31% NI, and 71% MI. Overall, a progressive decline in lesion size was observed in all model variants, but bioluminescent imaging showed some progression in size and new lesion formation in NI and MI mice. Lesions in DO and DI mice were mostly located on the peritoneal wall and mesentery/fat, while lesions in NI and MI mice were split relatively evenly between the peritoneal wall, mesentery/fat, and other locations like the bladder and wall of the uterus (Dorning et al., 2021).

#### Surgical Injection

The surgical injection murine model of endometriosis involves the injection of uterine fragments or decidualized uterine tissue through a surgical opening into the peritoneal cavity (Table 3). Minced tissue is taken from the uterine horns of a donor mouse, stripped of fat, muscle, and myometrium, and fragmented into smaller pieces usually less than 1.5 mm (Dabrosin et al., 2002; Burns et al., 2012; Jones et al., 2018). For decidualized tissue injections, decidualization is induced in ovariectomized females following the protocols discussed above in the intraperitoneal method section (Kim et al., 2020). In all studies, the uterine material is suspended in a solution that mimics the salinity and pH of the peritoneal cavity to maintain viability of the uterine tissue before injection into the peritoneal cavity; however, the suspension solution and volume (200 – 500 μl) varies among studies [e.g., saline (Alali et al., 2020), PBS (Burns et al., 2012; Jones et al., 2018), Hank’s buffered saline [HBS] (Li et al., 2016), and Basal Medium Eagle [BME] (Kim et al., 2014)]. The opening is then closed with sutures or wound clips, and then, at times, massaged to help spread the donor uterine material throughout the peritoneal cavity.

**TABLE 3:** Murine models of endometriosis: surgical injection method.

Strain	Induction age	D:R	Induction tissue	Donor primed	Rec. ovex’ed	E2	Tissue amount given	Stripped EM	Suspended uterine material	References
C57BL/6	D: 22–24 d R: 2–4 mo	1:1	Uterine fragments	PMSG	N	N	Ten 1 mm^3^ pieces	Y	Saline	Alali et al., 2020
C57BL/6J Ri	NM	NM	DT	E2 and P4	Y	Y	Ten biopsies	Y	NA	Peterse et al., 2018
C57BL/6-TG (UBC-GFP), WT	6–8 wk	1:1	UHs	PMSG	Y/N	Y	One 1.5 mm piece	Y	PBS	Jones et al., 2018
D: αERKO, C57BL/6-TG (GFP), IL6-KO R: αERKO, C57BL/6, IL6-KO	2–6 mo	1:1	UHs	PMSG	Y	Y	100 mg minced	Y	PBS	Burns et al., 2018
CD-1	6–8 wk	1:2	minced UH	PMSG	Y	Y	NM	Y	HBSS	Li et al., 2016
PR(Cre1) + Ptens(fl+), Ptens(fl+)	8 wk	Auto	minced UH	E2	Y	Y	60 mg	N	Eagle’s basal medium	Kim et al., 2014
D: αERKO, βERKO, C57BL/6 R: αERKO, βERKO, C57BL/6	NM	1:1 except αERKO 5:1	minced UH	PMSG	Y	Y	100 mg	N	PBS	Burns et al., 2012
C57BL/6 FVB/n	6–8 wk	1:1	minced UH	E2	Y	Y	One uterus	Y	Saline	Dabrosin et al., 2002

*Ovex, ovariectomy; EM, endometrium; E2, estrogen; P4, progesterone; D, donor; R, recipient; auto, autotransplantation of self-tissue; UH, uterine horn; DT, decidualized tissue; N, no; Y, yes; NM, not mentioned; NA, not applicable; h, hours; d, days; wk, weeks; mo, month; PMSG, pregnant mare serum gonadotrophin; PBS, phosphate buffered saline; HBSS, Hank’s buffered saline solution.*

In general, this model develops multiple lesion types. Typically, a lesion will form at the injection site and may approximate endometriosis formed in cesarean scars. Distal to the injection site, lesions progress through different stages and types depending on the time point in wild-type recipient mice: (1) 24 h after disease initiation, the uterine tissue has dispersed throughout the peritoneal cavity and is found lightly adhered at sites of attachment observed weeks later (Burns et al., 2018). This tissue is typically white to hemorrhagic, depending on the stage of angiogenesis. The peritoneal fluid lavage is mildly hemorrhagic. At this stage, the tissue histologically is disorganized lacking glands and stroma, but is full of red blood cell (RBC) and white blood cell (WBC) infiltrates. (2) 48 h after disease initiation, the uterine tissue is undergoing angiogenesis at the sites of attachment and the peritoneal fluid is hemorrhagic. The uterine tissue injected is more hemorrhagic than at 24 h, and the tissue is still histologically disorganized and resembles the lesions removed at 24 h, (3) 72 h after disease initiation, blood vessels are observable under a dissecting microscope, the peritoneal fluid is considerably less hemorrhagic, but the lesions, histologically, are still quite disorganized, hemorrhagic, and full of WBCs. At 72 h, the early lesions are beginning to attach more securely to the sites of attachment and are beginning to be encapsulated. (4) 3 weeks after disease initiation mature lesions are found that are cystic in appearance, exhibit organized structure, have distinct epithelial and stromal layers, and include hemosiderin macrophage deposits (Dabrosin et al., 2002; Burns et al., 2018). Lesions are typically light pink or tan, but hemorrhagic and white fibrotic lesions are also found. Lesions are found attached to the peritoneal/diaphragm wall, intestinal mesentery, gonadal and perivesical adipose tissue, behind the stomach/spleen, in the rectouterine cul-de-sac area, and on the uterine blood supply (Burns et al., 2018). Also mimicking human disease, lesions are not often found attached to the spleen, liver, or kidneys in wild-type mice (Burns et al., 2018).

The surgical injection model is similar to the intraperitoneal injection model in allowing for the study of the initial stages of the disease, which include angiogenesis, defective apoptosis, endometrial proliferation, inflammation, and chemotactic homing response (Sanchez et al., 2017). On average, in wild-type mice, this model produces 3–4 lesions per animal with variable sizes and weights (Burns et al., 2012, 2018). An advantage to this model is that lesion numbers are dependent on both recipient and donor genotype, reflecting the potential for gene and/or mechanistic pathway specificity in lesion development (Burns et al., 2012, 2018; Kim et al., 2014; Li et al., 2016; Jones et al., 2018; Peterse et al., 2018; Alali et al., 2020). Additionally, lesions are responsive to hormones and are altered by exposure to endocrine disrupting chemicals (Jones et al., 2018). A drawback of this model is that lesions may be difficult to locate unless a method of luminescence is employed. The variety of fragment sizes and the amount of tissue injected among study groups can make it difficult to compare experimental findings. With this model, reflux of the injected uterine tissue during closure of the surgical opening is possible.

#### Subcutaneous Placement Model

This model involves the placement of endometrial tissue in a subcutaneous pocket created in the ventral abdomen between the inner abdominal muscle and the peritoneal cavity (Table 4). Murine decidualized endometrium (Ferrero et al., 2017), murine uterine tissue (Wang et al., 2013), or human endometrial (heterologous) tissue (Wang et al., 2014) have been inserted into the pocket. A modified version of the engraftment model, the lesions developed are not affected by sutures. For this model in particular, immunocompromised mouse strains have been used to reduce the rejection of human tissue placed into the subcutaneous pocket. The lesions formed are smooth and well-defined with cyst-like structures (Wang et al., 2013) that display glands, inflammation, adhesions, and neo-angiogenesis (Ferrero et al., 2017). An advantage to this model is that small fragments of human endometrial or endometriotic tissue can be positioned in the murine system to study lesion growth and the effect of interventional treatments. However, in placing tissue outside the peritoneal cavity, this model poorly recapitulates the pathogenesis and pathophysiology of human disease. Additionally, the reduction of immunocompetence in the heterologous version of this model may alter the hallmark inflammatory response observed in human endometriotic lesions.

**TABLE 4:** Murine models of endometriosis: subcutaneous placement method.

Strain	Induction age	D:R	Induction tissue	Donor primed	Rec. ovex’ed	E2	Tissue amount given	Stripped EM	References
C57BL/6 B6N-Tyr(c-BRD)/BRDCr-Crl	8 wk	NM	DT	NM	N	N	3–5 mm^3^ pieces	N	Ferrero et al., 2017
D: CMV-Luc and NOD/SCID	8 wk	1:2	UHs	N, in estrous	N	N	Five 2 mm punches	N	Wang et al., 2013
BALB/c	4–5 wk	NM	Human endometrial cells	NA	N	N	400 EECs mass	NA	Wang et al., 2014
D: K-ras*^G12V/+^*/Ah Cre^+/+^/ ROSA26R-LacZ^+/+^ R: C57BL/6	NM	4:1	DT in Matrigel	BNF into UH	Y/N	Y/N	One UH	N	Cheng et al., 2011

*Ovex, ovariectomy; EM, endometrium; E2, estrogen; D, donor; R, recipient; UH, uterine horn; UHs, two uterine horns; DT, decidualized tissue; HS, human samples; N, no; Y, yes; NM, not mentioned; NA, not applicable; wk, week; EEC, endometrial epithelial cells; BNF, beta-naphthoflavone.*

#### Spontaneous Translocation

Spontaneous retrograde translocation of endometrium via a surgically modified reproductive tract recently introduced a new category of murine model of endometriosis (Table 5; Wilson et al., 2020). Wilson et al. (2020) reported the induction of endometriosis in a genetically modified CD-1 strain via retrograde translocation of endometrium harboring *Arid1a* and *Pik3ca* modifications. Loss of ARID1A expression coupled with over-expression of oncogenic PIK3CA mutation in the endometrium of these mice was previously demonstrated to result in adenomyosis-like invasion of the endometrium into the uterine myometrium (Wilson et al., 2019). In order to evaluate the use of this genetically engineered mouse strain in modeling endometriosis, a surgical incision was made at the utero-tubal junction followed by salpingectomy, to allow mutated endometrial epithelial cells access to the peritoneal cavity. The ovaries were not removed in order to avoid an exogenous hormone requirement for disease induction and to allow the investigation of ovarian endometriosis phenotypes. In over 50% of genetically modified mice undergoing the surgical procedure, a variety of lesions were grossly observed and histologically confirmed, including ovarian and peritoneal phenotypes (Wilson et al., 2020). Interestingly, lesions were not observed in three of the eight wild-type CD-1 mice that underwent the same procedure.

**TABLE 5:** Murine models of endometriosis: spontaneous translocation method.

Strain	Induction age	D:R	Induction tissue	Donor primed	Rec. ovex’ed	E2	Tissue amount given	Stripped EM	Suspended uterine material	References
Mutated CD-1	6 wk	Auto	Mutated endometrial epithelial cells	NA	N	N	NA	NA	NA	Wilson et al., 2020

*Ovex, ovariectomy; EM, endometrium; E2, estrogen; D, donor; R, recipient; auto, autotransplantation of self-tissue; N, no; NA, not applicable; wk, week.*

This model provides an immunocompetent, hormonally intact, semi-autologous induction of endometriosis, and represents the most accurate recapitulation of retrograde translocation of endometrium described to date. However, it is unclear if endometrial decidualization similar to human endometrium occurs in the genetically modified endometrium. Vaginal bleeding is a side effect of this model, but it was not mentioned if this side effect was due to the ARID1A and PIK3CA mutations or will be the case in all animals. Additionally, the long term patency of the utero-tubal incision is unknown and may impact the ability of the model to allow multiple seeding events of endometrium into the peritoneal cavity (Dodds et al., 2017). This model is unlikely to be useful for longitudinal studies of endometriosis due to adverse side effects after the procedure, including vaginal bleeding, a distended abdomen, and death around 17 weeks post-procedure (Wilson et al., 2020). Finally, it is currently unknown to what extent the completely penetrant genetic modifications in this mouse model reflect the nature or dose of somatic mutation that predisposes retrograde menstruated human endometrium to implant and grow in the peritoneal microenvironment.

### Luminescence

Luminescence allows the resolution of endometriotic lesions that are difficult to detect macroscopically and has the potential to offer a non-invasive evaluation of disease. Here, we describe the variety of strategies that have been evaluated for this purpose in murine models of endometriosis (Table 6).

**TABLE 6:** Murine models of endometriosis: lesion analysis.

Study length	Luminescence method	*In vivo* imaging	Necropsy cycle phase controlled	Control type	Control lesion #	Control lesion size	References
7, 21, 42 d	Luciferase/GFP	PhotonIMAGER	N	Sham: saline injection only	NA	NA	Dorning et al., 2021
4 wk	u-GFP	N	N	Sham: suture only	NA	NA	Chen et al., 2021
20 d	N	N	N	Vehicle: polyethylene glycol	3 – SP	∼18 mg; ∼0.028 cm^3^	Santorelli et al., 2021
4 wk	N	IVIS	E Tx	Vehicle: 0.1% DMSO	4 – SP	5 ± 1 mg; 5 mm^2^	Sharma et al., 2021
3 wk	mCherry	IVIS	E Tx	Vehicle: 5% glucosaline	2 – 3 SP	NM	Tejada et al., 2021
12–20 wk	N	N	N	Vehicle: PBS	1.4 ± 0.5	60 mg ± 0.02	Ono et al., 2021
∼17 wk	GFP	N	NM	CD-1	NM	NM	Wilson et al., 2020
1, 2, 3, 7, 28 d	N	N	NM	Sham: PBS injections	2 – SP	NM	Symons et al., 2020
15, 30, and 60 d	N	N	Y	NM	1 – SP	NM	Mishra et al., 2020
2 mo	N	N	NM	Vehicle: DMSO	4 – SP	NM	Li et al., 2020
2 wk	N	N	E Tx	Vehicle: saline	NM	NM	Kim et al., 2020
14, 28, 42, and 56 d	N	N	Y	Sham: HBSS IP, Vehicle: DMSO	NM	∼4 mm	Fattori et al., 2020
NM	N	N	NM	Sham: fat pad	NM	NM	Alali et al., 2020
2 wk	N	Ultrasound	E Tx	Sham: fat pad, Vehicle: E2	2 – SP	18 mg; 20 mm^3^	Chang et al., 2020
0, 7, 14, 21 d	N	N	E Tx	NM	2 – SP	NM	Hattori et al., 2020
6 wk	N	N	E Tx	NM	NM	NM	Woo et al., 2020
1, 2, 4 wk	N	N	N	Sham: suture only	2 – SP	NM	Hayashi et al., 2020
3–4 wk	N	N	N	Vehicle: saline	NM	65 mg ± 0.2	Yan et al., 2019
4 wk	Cre- ZsGreen	N	Y	WT to WT	NM	NM	Tal et al., 2019
5 wk	N	Ultrasound	E Tx	NA	1 – SP	NA	Peyneau et al., 2019
15 d	N	N	N	NM	NM	∼3 mm	Mattos et al., 2019
4 wk	mCherry	Y	E Tx	NA	NA	NA	Martinez et al., 2019
3 wk	GFP	N	NM	Vehicle: water PO	2.06 ± 0.32	NM	Horne et al., 2019
4 wk	GFP	N	E Tx	Vehicle: saline	NM	NM	Forster et al., 2019
3 wk	N	N	E Tx	Sham: suture only, Vehicle: aspartame	1 – SP	3 mg; 3.5 mm^3^	Chadchan et al., 2019
7, 14, 21, 28 d	GFP	N	E Tx	WT control mice	2 – SP	10 mm^2^	Sekiguchi et al., 2019
7, 14, 28, 42 d	N	N	N	NM	NM	NM	Yuan et al., 2018
1 wk	N	N	E Tx	NM	∼5	NM	Peterse et al., 2018
6 wk	GFP	N	Y/E Tx	Vehicle: NM	3 ± 2	11 ± 2 mg	Jones et al., 2018
24, 48, 72 h or 3 wk	N	N	E Tx	Vehicle: corn oil	2	∼100 mg	Burns et al., 2018
2 wk	GFP	N	N	Vehicle: PBS	1.00 ± 0.26	1.66 ± 0.027 mm^3^	Sanchez et al., 2017
20 d	mCherry	Carestream *in vivo* FX-PRO	N	IHC of human lesions	1	NM	Ferrero et al., 2017
3 wk	N	N	Y	Sham: saline injection	NA	NA	Dodds et al., 2017
5 wk	N	N	N	Vehicle: PBS	1–5	10 mm^2^	Ruiz et al., 2016
4, 8, 12, 16, 20, 24 d	N	N	E Tx	None	3–4 in E2 group	150–250 mm^3^ E2 group	Li et al., 2016
8 wk	N	N	N	NM	1.4 ± 0.3	16.9 ± 5.6 mm^3^	Heard et al., 2016
4 wk	N	N	E Tx	Vehicle: DMSOx2 wkly	4.5–5	∼78 mg	Uegaki et al., 2015
31 d	N	N	Y	Sham: sutures only	2 – SP	NM	Cohen et al., 2015
3, 7, 14 d	N	N	Y	Hormonally intact animals	2 – SP	NA	Zhao et al., 2014
16 wk	N	N	N	Vehicle: DMSO in sesame oil	NM	19.6 mm^2^	Naqvi et al., 2014
4 wk	GFP	N	NM	NM	NM	NM	Machado et al., 2014
3, 4, 5 wk	N	N	E Tx	Sham: E2/no E2	NA	NA	Liao et al., 2014
3 wk	N	N	Y, estrus	Sham: sutures only, Vehicle: EtOH/PBS	6 – SP	2.5 ± 0.6 mm^3^	Kumar et al., 2014
4 wk	N	N	E Tx	Vehicle: 30% captisol/wk	4.50 ± 0.34	NM	Kim et al., 2014
3 wk	MacGreen GFP	N	E Tx	NM	2.06 ± 0.32	NM	Greaves et al., 2014
26 d	N	N	NM	Non-pregnant group	2 – SP	15.1 ± 2 mg	Cohen et al., 2014
4 wk	Luciferase	IVIS	N	Vehicle: saline	1 – SP	11 mg; 8 mm^3^	Wang et al., 2014
2–3 wk	GFP	N	Y, estrus	Vehicle: corn oil	NA	NA	Pierzchalski et al., 2014
2 wk	N	N	E Tx	WT mice	∼5 ± 0.5	15-17 mg; 2.5 mm	Tomio et al., 2013
4 wk	N	N	E Tx	Vehicle: 1% DMSO	5.8 ± 0.9	65.6 ± 14.6 mg; 50.3 ± 12 mm^2^	Takai et al., 2013
8 wk	N	MRI/exploratory laparotomy	E Tx	IgG isotype controls	5 – SP	B6: 205.9 ± 38.86 mm^3^ CD1: 105.6 ± 14.2 mm^3^	Silveira et al., 2013
2 wk	GFP	N	Y, estrus	Vehicle: corn oil	3.14 ± 0.5	18.4 ± 4.4 mm^3^	Wieser et al., 2012
3 wk	N	N	Y, estrus	Vehicle: corn oil	1.8–2.1	1 mg	Burns et al., 2012
2 wk	GFP	N	E Tx	Vehicle: corn oil	2 – SP	65–75 mg; 50–60 mm^3^	Wilkosz et al., 2011
4 wk	N	N	N	Vehicle: saline	3 – SP	25.1 ± 6.2	Ricci et al., 2011
16 wk	N	N	N	Vehicle: DMSO	2 – SP	28 ± 3 mg; 60 mm^2^	Kulak et al., 2011
3 wk	N	N	E Tx	Vehicle: NM	NM	25.3 ± 21.4 mm^2;^ 23.8 ± 16.9 mg	Itoh et al., 2011
3 wk	N	N	E Tx	Vehicle: oil	1 – SP	∼22 mm^2^	Cheng et al., 2011
NM	GFP	N	N	Sham: suture only	NA	NA	Becker et al., 2011
3 wk	N	N	E Tx	Vehicle: water gavage	1.2 ± 0.1	NM	Pittaluga et al., 2010
6 wk	N	N	E Tx	Sham: fat pads, Vehicle: DMSO	2.6 ± 1.1	22.3 ± 9.6 mm^2^	Lu et al., 2010
6 or 8 wk	N	N	N	Collection of a UH at induction	NA	NA	Kusakabe et al., 2010
4 wk	N	Ultrasound	N	None	2 - SP	NA	Körbel et al., 2010
40 h	Celltracker green	N	E Tx	WT w/PBS treatment	8.57 ± 0.39	NM	Jensen et al., 2010
4 wk	N	N	N	Vehicle: saline	3 – SP	310 ± 80 mm^3^	Bilotas et al., 2010
2 wk	Fluorescent dye	N	N	Vehicle: PBS	NM	65 ± 20 mg	Altan et al., 2010
24, 48, 96 h	N	N	N	Sham: white adipose tissue	NA	NA	Umezawa et al., 2009
NM	N	N	N	Sham: NM	NA	NA	Lee et al., 2009
24 d	N	N	E Tx	Sham: NM, Vehicle: saline	NM	68.89 ± 7.2 mg	Chen et al., 2009
12 d	N	N	N	Vehicle: PBS	NM	12 ± 1 mg	Bacci et al., 2009
1–4 wk	N	N	N	Vehicle: PBS	NM	NM	Fainaru et al., 2008
10 d	eGFP	N	N	NM	NM	NM	Nowak et al., 2008
7 d	N	N	N	Vehicle: NM	3	NM	Matsuzaki et al., 2008
4 wk	GFP	N	E Tx	Vehicle: NM	7 – SP	NM	Becker et al., 2008
23 d	N	N	E Tx	Sham: PBS injection, Vehicle: NM	2.8 ± 1.2	12.6 ± 1.5 mg	Yoshino et al., 2006
2 wk	GFP	N	E Tx	Vehicle: corn oil	2.2 ± 0.5	1.68 ± 1.44 mg	Hirata et al., 2005
4 wk	N	N	E Tx	Vehicle: methylcellulose	7 – SP	5.8 ± 2 mm^2^	Efstathiou et al., 2005
4 wk	N	N	N	NM	None	NA	Cao et al., 2004
1 and 3 wk	N	N	E Tx	Control virus in PBS	NM	NM	Dabrosin et al., 2002
3 wk	N	N	E Tx	NM	NM	2.66 ± 0.45 mg; 25.64 ± 2.87 mm^2^	Somigliana et al., 1999
3 wk	N	N	E Tx	Vehicle: corn oil	3 – SP	3.6 ± 0.22 mm	Cummings and Metcalf, 1995

*h, hour; d, day; wk, week; mo, month; N, No; Y, Yes; NM, not mentioned; NA, not applicable; PO, oral; WT, wild-type mice; SP, surgically placed; UH, uterine horn; E Tx, exogenous treatment; GFP, green fluorescent protein; DMSO, dimethyl sulfoxide; EtOH, ethanol; IgG, immunoglobulin G; PBS, phosphate buffered saline.*

#### Green Fluorescence

The first use of luminescence in a murine model of endometriosis was reported by Hirata et al. (2005). A transgenic mouse ubiquitously expressing GFP on the C57BL/6 background was used as donor tissue. Minced uterine pieces were injected (18-gauge needle) into the peritoneal cavity of a recipient ovariectomized wild-type mouse receiving either no estrogen (control) or estrogen weekly. At necropsy, lesions were easily located using a fluorescent lighting system. The ability to detect GFP expressing tissue allows for easier assessment in regression studies where compounds are expected to reduce or destroy lesions. Histological analysis to examine GFP using an anti-GFP antibody reveals a clear difference between donor and recipient tissue, allowing a more detailed assessment of endometrial-mesothelial interactions in studies of lesion formation. Since this initial report, several more targeted GFP transgenic models have been developed.

The MacGreen model has been used to study macrophage function in lesion development (Sasmono et al., 2003). MacGreen mice were engineered to express enhanced GFP in macrophage and monocyte cells stimulated by Colony Stimulating Factor-1 (CSF-1) (Chen et al., 2015). This model was employed for the study of inflammatory pathways involved in lesion induction, but limited to CSF-1 expressing cell types. Importantly, MacGreen mice evidence compromised fertility of undetermined etiology. The possibility for endometrial dysfunction as the cause of subfertility dissuades use of this transgenic strain as the endometrial donor in modeling endometriosis.

To study cell fusion events in lesions, Tal et al. (2019) used β-actin-Cre mice (expressing Cre recombinase directed by the human beta actin gene promoter) crossed with ZsGreen mice [containing a targeted mutation of the *Gt(ROSA)26Sor* locus with a loxP-STOP-loxP-ZsGreen1 cassette to prevent EGFP transcription (Madisen et al., 2010; Tal et al., 2019)]. The ZsGreen/LoxP mice produced offspring with high expression of ZsGreen (Nakamura et al., 2013). When the β-actin-Cre host received endometrium from the ZsGreen/LoxP donor, the fused cells expressed eGFP, revealing that cell fusion occurs in endometriotic lesions and that bone marrow derived cells participate in these cell fusion events (Tal et al., 2019).

The Ubiquitin C-GFP (UbC-GFP) model has also been used to study hematopoietic cells by allowing *in vivo* leukocyte tracking and hematopoietic cell differentiation in a murine model of endometriosis (Schaefer et al., 2001). Chen et al. (2021) used UbC-GFP mice to analyze mesenchymal stem cell differentiation and PD-1 expression after induction of endometriosis and demonstrated that bone marrow derived cells bind to lesions. Thus, localization of tissue or cells is critically important to track various stages of development in models of endometriosis.

If genetically modified mice are not available, CellTracker Green (Thermo-Fisher) is a fluorescent dye that can be used for short term studies. The dye passes through the cell membrane and becomes a non-permanent fluorescent product with decreasing fluorescent intensity per mitotic event and lasting up to 72 h (Lilius et al., 1996). Jensen et al. (2010) used CellTracker Green to monitor the initiation of lesion formation in the first 40 h after induction. Uterine fragments were minced, homogenized, labeled with CellTracker Green, and injected (18-gauge needle) into the peritoneal cavity of recipient mice (Jensen et al., 2010). After 40 h, mice were euthanized and lesions within the peritoneal cavity were located using a fluorescence stereomicroscope.

#### Red Fluorescence

Green fluorescent protein is a useful tool to detect lesions at necropsy (Becker et al., 2006). However, with a short emission wavelength of 510 nm, GFP does not penetrate tissue well (Ferrero et al., 2017), thereby limiting its utility for longitudinal studies predicated on *in vivo* imaging of fluorescent lesions. mCherry, a red fluorescent reporter, is a brighter fluorophore with an emission spectrum of 550–650 nm (Piatkevich and Verkhusha, 2011). Importantly, due to the higher emission wavelength and greater photostability, mCherry has deeper tissue penetration (Ferrero et al., 2017) allowing for more accurate *in vivo* fluorescent imaging. Several studies report the use of a mCherry expressing adenoviral vector to infect either mouse or human tissue samples prior to implantation in donor mice (Ferrero et al., 2017; Martinez et al., 2019; Tejada et al., 2021). The mCherry signal was found to be strong and viable for at least 20 days (Ferrero et al., 2017). A drawback to this model is that the transfected fluorescent signal fades over time, and therefore does not allow for serial *in vivo* detection. A constitutively expressing mCherry mouse strain exists (Fink et al., 2010), but requires cryorecovery. With this mouse model, lesion location affects the ability to accurately detect and monitor lesion size and final location. For example, lesions located near the dorsal aspect of the peritoneal cavity are more difficult to detect than lesions near the ventral abdomen (Ferrero et al., 2017). With the intraperitoneal injection, surgical injection, or spontaneous translocation models, lesions may be located anywhere in the peritoneal cavity. Incomplete fluorophore detection can lead to errors in quantification of accurate lesion size and/or number within a recipient mouse, especially for lesions located in the dorsal abdomen. The signal-to-noise ratio can be optimized by using albino or nude mouse strains (Ferrero et al., 2017).

#### Luciferase

An alternative to fluorescence for *in vivo* imaging purposes is presented by bioluminescence-based strategies. When transgenic mice or tissues expressing firefly luciferase under the ubiquitin C promotor (UbC-Luc) are treated with luciferin, a detectable bioluminescent signal is expressed (Becker et al., 2006). In the context of a mouse model of endometriosis, transplanting uterine tissue from a donor UbC-Luc mouse into a wild-type recipient mouse and injecting luciferin subcutaneously, into the tail vein, or into the peritoneal cavity of the recipient prior to *in vivo* imaging will result in bioluminescence of lesions (Wang et al., 2013; Dorning et al., 2021). Lesion size and weight can be correlated with signal intensity (Wang et al., 2013; Dorning et al., 2021). Unfortunately, as with fluorescence, coat color of the mouse strain affects the *in vivo* detection of luminescence, with black coat reducing luminescence by ∼10 fold (Becker et al., 2006). This limits selection to albino, nude mouse strains, or backcrossing to the Tyrosinase negative (Tyr) Bl6 mice which have a white coat color. The method of luciferin injection also affects lesion imaging. Tail vein and subcutaneous injections allow luciferin to traverse intravascularly, but if lesions have not undergone neoangiogenesis, such as the day after disease induction, no fluorescent signal will be seen (Becker et al., 2006).

#### Non-luminescent Methods

Ultrasound represents a non-invasive method for repeat visualization of lesions without the requirement for luminescence (Laschke et al., 2010). Implant viability and size of lesions a few days after induction (Peyneau et al., 2019), and lesion growth via weekly volume measurements (Körbel et al., 2010; Chang et al., 2020) using high-frequency ultrasound imaging systems have been reported. For ultrasonography, the mouse is anesthetized and placed on a heat source. Ultrasound gel is applied to the abdomen, and a two-dimensional view of the implant is acquired as the probe moves across the abdomen. For this study, only one 5 mm graft was placed, but the implant size was calculated as volume in millimeters (Peyneau et al., 2019). Implant viability was determined by the visualization of cyst-like endometrial glands and vascularized endometrial stroma at image analysis (Laschke et al., 2010). This method is well suited to the engraftment model because the initial size and location of the donor tissue is known compared to the variability in lesion size and location in the injection models of endometriosis. Additionally, respiratory movement and intestinal peristalsis makes lesions that adhere to certain areas, i.e., the diaphragm or intestines, indistinguishable from other organs (Laschke et al., 2010). MRI for *in vivo* volumetric measurements of endometrial implants has also been described (Silveira et al., 2013).

### Commonly Used Controls

The use of proper controls with murine models of endometriosis not only helps to solidify findings but is also critical for establishing a baseline for comparison and data interpretation. Controls should be selected based on experimental design, dosing strategies, and experimental endpoints (i.e., timing of lesion removal).

#### Sham

There are two types of sham surgeries widely used in the murine model of endometriosis. The first sham surgery performs the procedure without introducing tissue, but places a suture in the peritoneal cavity where tissue would have been placed and then closes the incision (Chen et al., 2009; Lee et al., 2009; Kumar et al., 2014; Liao et al., 2014; Cohen et al., 2015; Chadchan et al., 2019; Chang et al., 2020; Hayashi et al., 2020). The second sham surgery uses sutured, or injected, fat pads into the peritoneal cavity (Umezawa et al., 2009; Lu et al., 2010; Alali et al., 2020; Chang et al., 2020). These designs provide readout regarding the effects of the surgical procedure on the induction or course of endometriotic lesions as well as the impact of the procedure on the health of the animal.

#### Vehicle

Dimethyl sulfoxide (DMSO) is frequently used as a vehicle to deliver treatment (Lu et al., 2010; Kulak et al., 2011; Takai et al., 2013; Naqvi et al., 2014; Uegaki et al., 2015; Li et al., 2020; Sharma et al., 2021). For long term studies, repeated doses with a lower percentage of DMSO is preferred.

Many studies also use oil as a solvent for oral gavage, food, and SQ administration. For example, in order to manipulate hormone levels in ovariectomized mice, estradiol is commonly dissolved in ethanol and mixed with corn oil for subcutaneous injection (Wilkosz et al., 2011; Chang et al., 2020). Oil, if used for intraperitoneal administration, can cause an inflammatory response in mice, with severity depending on the type of oil used. Mineral and peanut oil have the highest inflammatory response while corn and olive oil produce less of a reaction (Alsina-Sanchis et al., 2021). Inflammation caused by oil injections may interfere with immune cell recruitment and the overall immune response (Alsina-Sanchis et al., 2021). Analysis of the immune response to endometriosis may be confounded by the inflammatory response elicited by the presence of oil in the peritoneal cavity (Cummings and Metcalf, 1995; Hirata et al., 2005; Cheng et al., 2011; Kulak et al., 2011; Wilkosz et al., 2011; Burns et al., 2012, 2018; Wieser et al., 2012; Naqvi et al., 2014; Sharma et al., 2021).

Other vehicles used in the delivery of drugs and agents in murine models include PBS (Bacci et al., 2009; Altan et al., 2010; Sanchez et al., 2017; Symons et al., 2020), water (Pittaluga et al., 2010; Horne et al., 2019), saline (Chen et al., 2009; Bilotas et al., 2010; Ricci et al., 2011; Dodds et al., 2017; Forster et al., 2019), polyethylene glycol (Santorelli et al., 2021), 0.1% bovine serum albumin/PBS (Yoshino et al., 2020), ethanol/PBS (Kumar et al., 2014), captisol (Kim et al., 2014), and IgG isotype (Silveira et al., 2013).

#### Genotype

When using various gene specific mouse knockout, knockin, or conditional strains of mice [e.g., ESR1^–/–^ mice (Burns et al., 2018)], a wild-type donor to wild-type recipient is needed as a control. A wild-type to wild-type control allows for the comparison of results from a non-genetically engineered model to one with the desired genetic alteration. Appropriately incorporated, a genetic control sets conditions for correct interpretation of the role of various genes, cell types, and/or responses to treatments.

## Critical Challenges in Developing a “Best Fit” Mouse Model for Endometriosis

### Genetic Background

The choice of strain for a murine model often requires a decision on the importance of genetic background in modeling endometriosis. Mice may either be inbred or outbred. Inbred mice are genetically homogeneous and offer little variation or heterozygosity (<1%). The inbreeding process can lead to the fixation of allelic states, interactions, and responses (Tuttle et al., 2018) that may bring about the development of undesirable traits (i.e., malocclusion) or different responses to methods or medications than a more diverse group of mice may exhibit (Yoshiki and Moriwaki, 2006). Importantly, though, the genetic conformity of the inbred strains allows for donor uterine tissue to be implanted into a recipient mouse without fear of tissue rejection or the need for anti-rejection drugs (Tuttle et al., 2018). Outbred mice are genetically heterozygous and better reflect a diverse population due to higher genetic variation (The Jackson Laboratory, 2006); however, tissue rejection is common and/or lesions are not maintained in the mice [e.g., CD-1 endometriosis (Li et al., 2016)]. Despite the lack of genetic diversity to reflect the human condition, inbred mice are extremely useful in designs that seek to leverage the standardization and extensive molecular annotation of inbred lines.

If an inbred line is used, strain can represent an additional caveat to consider. For example, C57BL/6 and BALB/c mice differ in their immune response – composition, timing, and location of cytokine release are different in relation to specific immune cues (Dodds et al., 2017). The C57BL/6 mice are more Th1 immune responsive while the BALB/c mice have a more Th2 dominant response. The innate immune response of macrophages is different between the two strains (Watanabe et al., 2004). Dodds et al. (2017) found that BALB/c mice are 2.7 times more likely to develop cystic lesions than wild-type C57BL/6 mice. Recent evidence implicates a Th2 dominant pathway in the incomplete clearance of retrograde menstruum in endometriosis pathogenesis (Liang et al., 2019), though data implicating Th1 or Th2 dominance in endometriosis are generally inconclusive.

### Immunocompetence

Immunocompetence is a challenge when using human uterine tissue or human endometriotic tissue in a murine model. Immunocompromised mice may not reflect the environment within the human peritoneal cavity, and the results of the experiment may not accurately reflect human disease initiation. It is also important to understand the immune response of the mouse strain being used. Immunocompromised mice lack different innate and adaptive immune cell populations that may lead to differing findings depending on the cell type that is absent or dysregulated. Choosing a strain with a standardized and well characterized background for human tissue studies in immunocompromised mice is advisable.

### Estrogen Dependence

Estrogen dependence is a molecular hallmark of endometriosis pathophysiology. In women, a lack of estrogen halts lesion progression and growth and may treat some pain symptoms, but lesions do not disappear (Dlugi et al., 1990; Taylor et al., 2017; Vercellini et al., 2019; Poulos et al., 2021). Early models of ovariectomized mice coupled with exogenous estrogen administration were instrumental in highlighting the potentiating role of estrogen in endometriosis. However, the exogenous administration of estrogen can have off-target and even confounding effects in murine models. Supraphysiologic estrogen dosing can lead to systemic changes such as uterine growth (Edwards et al., 2013), cell proliferation (Groothuis et al., 2007), and immune system alterations that reduce NK cell activity (Seaman and Gindhart, 1979), inhibit B cell development, reduce T cell populations, and induce monocyte apoptosis (Lang, 2004). Ovariectomy introduces variables of surgery, convalescence, and ovarian hormone depletion and prevents study of endometriosis impact on fertility. When hormonally intact mice are used, lesions are exposed to the full complement of the hypothalamic pituitary ovarian axis, which is more representative of the human condition. Importantly, with this model, mice should be euthanized in the same stage of the estrous cycle for normalization of data. Decisions regarding sex-steroid exposure are therefore a key consideration in model development.

### Vehicle Selection

Oils are a commonly used vehicle for the delivery of therapeutic agents in murine models. Oils frequently used as vehicles include corn oil, olive oil, peanut oil, and mineral oil (Alsina-Sanchis et al., 2021). The use of oil as a vehicle can confound model read-out particularly in the case of intraperitoneal injection. In the peritoneal cavity, the presence of oil can reduce the number of resident macrophages and lead to higher and altered levels of inflammation (Alsina-Sanchis et al., 2021). Injection of oil into the peritoneal cavity may also lead to morphological changes in the greater omentum and the intestinal mesentery (Alsina-Sanchis et al., 2021). Oil injected into the peritoneal cavity at high frequency (e.g., daily) may incompletely clear from the peritoneal cavity leading to a prolonged inflammatory response (Alsina-Sanchis et al., 2021). Given the role of an intact immune system in endometriosis pathophysiology, the use of oil in the peritoneal cavity may complicate data interpretation.

### Reproductive Tract Considerations

Humans and mice differ in the embryologic development and subsequent anatomy of the reproductive tract. Mouse vaginal epithelium is thought to derive from Mullerian duct epithelium while human vaginal epithelium may be replaced by FOXA1 positive urogenital sinus epithelium (Cunha et al., 2019). Mice exhibit a closed reproductive system with tightly coiled oviducts that open into the bursal space while the human reproductive system is open to the peritoneal cavity (Rendi et al., 2012). This open reproductive system in humans allows endometrial cells entry into the peritoneal cavity through “retrograde menstruation” at each episode of menses, thereby providing multiple seeding events (Sampson, 1927). Usually murine models of endometriosis feature one seeding event. To truly mimic human disease, multiple seeding events should be present, but multiple invasive procedures can induce increased inflammation, surgical complication, and an altered immune state within the peritoneal cavity (Dodds et al., 2017). Wilson et al. (2020) innovatively approached this problem in a model involving the spontaneous translocation of endometrial cells into the peritoneal cavity following salpingectomy and opening of the utero-tubal junction. While this model seems to convert a closed reproductive tract to an open one, the mice experience severe side effects (i.e., vaginal bleeding, distended abdomen, early death) as a result of the endometrial genetic modifications which limits the longitudinality of the model.

Mice experience a 4–5-day estrous cycle that begins at ∼26 days old (Ajayi and Akhigbe, 2020) and features a proestrus, estrus, metestrus, and diestrus phase (Sato et al., 2016). In mice, ovulation occurs in both ovaries each cycle (Sato et al., 2016). In contrast, humans experience a menstrual cycle that begins at puberty, lasts ∼28 days, and includes menstrual, proliferative, and secretory phases (Ajayi and Akhigbe, 2020). These early developmental and cycle dynamic differences present obvious challenges in using a murine system to model human gynecologic diseases such as endometriosis.

### Lesion Subtyping

Another challenge to developing a “best fit” model is that human lesions are not fully subtyped and characterized. This challenge makes it difficult to develop models that recapitulate all types of endometriotic disease. Efforts are being taken to phenotype and subphenotype lesions (Colón-Caraballo et al., 2019); however, we still may not know all of the different types and stages of lesions found in human disease. A proteo-genomic database stratified by lesion location, size, and appearance in the human condition would clarify disease subphenotypes for modeling this enigmatic disease.

### Visualization of Lesions

While challenges exist with the penetration of fluorescent signal through tissue and hair, and lesion placement in the cavity (i.e., GFP and luciferase), the use of fluorophores with longer wavelength emission spectra (i.e., TdTomato and mScarlet) may optimize signal-to-noise ratios for longitudinal *in vivo* studies designed to track lesion progression and/or regression (Piatkevich and Verkhusha, 2011; Bindels et al., 2017).

### Model Reporting

The final, and, perhaps, most important challenge for endometriosis research is the potential for incomplete reporting of promising models in the setting of negative experimental results. Negative or inconclusive results are rarely published, and this may result in groups expending time and resources developing a murine model that another group may have already developed but failed to publish (Malvezzi et al., 2020). A potential solution to avoid redundancy in model development is the establishment of a database or public forum for groups to post negative results for consultation, critique, and input from other groups. Increased emphasis among journals to publish both positive and negative results will more widely promote promising model features toward achieving a “best-fit.”

## Key Points for Establishing the “Best-Fit” Murine Model of Endometriosis

### Spontaneous Attachment, Growth, and Maintenance of Endometriotic Lesions

Endometriosis is histologically defined by the presence of endometrial glands and stroma in non-uterine locations, and, in some lesions, the presence of hemosiderin-laden macrophages (Hsu et al., 2010). Lesions are also characterized by dense vascularization (Sekiguchi et al., 2019) innervation, and fibrosis (Mishra et al., 2020). The histology of the endometriotic lesions produced by the model is important when selecting a method of disease induction. Pelch et al. (2010) were able to demonstrate that lesions from mice have aberrant gene expression profiles that mirror patterns observed in human lesions suggesting that lesions developed in mice can lead to mechanistic understanding of endometriotic lesions in humans. Ideally, the model recapitulates the spontaneous attachment of endometrial cells/tissue to the mesothelium, lesion development and growth, and host inflammatory reaction to lesions.

Human lesions are found in a variety of locations, sizes, colors, and may be superficial or deep infiltrating endometriotic lesions. A mouse model that recapitulates all of these disease subtypes may not be feasible, but several murine models do reasonably well in reflecting lesion diversity. Since the initial amount of donor uterine tissue used correlates with subsequent growth and angiogenesis density (Körbel et al., 2010), it is critical to specify the tissue type, tissue amount, endometrial priming methods, and induction method used in the model. For example, intraperitoneal and surgical injection methods reviewed vary widely in the amount of uterine/endometrial tissue injected.

The menstrual cycle phase of endometrium at the time of implantation in the peritoenum is a challenging feature to model when using a non-menstrual species such as the mouse. Decidualization of murine endometrium can be achieved by several approaches with oil injection into the uterus providing more decidualized tissue than internal scratching (Ferrero et al., 2017). Alternatively, menstrual endometrium can be developed in donor mice through validated hormonal programs (Greaves et al., 2014). In the baboon, endometriosis was more efficiently induced with menstrual versus luteal phase endometrium, as evidenced by the higher number and larger surface area of endometriotic lesions and the more advanced diagnosed stages of endometriosis in animals receiving menstrual tissue (D’Hooghe et al., 1995). The use of menstrual endometrium seems to not only approximate human disease pathogenesis, but also enhances the efficiency of lesion development.

A confounding effect of the induction method on the pathogenesis and/or pathophysiology of lesions represents a key consideration in model development and use. Subtle molecular or cellular changes in endometriotic lesions may result from the surgical procedure required to induce disease (Dodds et al., 2017). As an example, in the surgical engraftment model, lesions in close proximity to sutures may evidence inflammatory cell recruitment and angiogenesis as a result of foreign body reaction to the sutures rather than the lesions (Wilkosz et al., 2011; Wang et al., 2013).

### Immune Regulation and Hormones

The pathogenesis of endometriosis is believed to involve sex-steroid hormone and immune-mediated pathways. The peritoneal fluid milieu in women with endometriosis is significantly different from that of unaffected women. Women with endometriosis have increased macrophage activation, secretion of growth and angiogenic factors, and increased presence of reactive species, contain pro-inflammatory conditions, and may have dysfunction of natural killer lymphocyte activity (Cohen et al., 2015). This altered peritoneal microenvironment is difficult to recreate in a mouse model, but, because of the active involvement of the immune system in disease initiation, the use of immunocompetent strains is preferred.

Also critical for lesion growth is hormonal regulation. While mice do not menstruate, their cyclical hormonal profile is similar to humans and allows for the role of hormones and hormonal regulation to be studied in lesion responses. The induction of endometriosis does not disrupt estrous cycling (Jones et al., 2018; Santorelli et al., 2021), but lesions, like uterine tissue, are responsive to hormonal changes. For example, gene expression and cell proliferation increase with estrogen (Burns et al., 2012; Jones et al., 2018). As with human studies of endometriosis, cycle phase is an important variable to consider in the interpretation of molecular signatures of the endometrium or lesions in murine models. Control for this variable can be approached by allowing natural estrous cycling in models with intact ovaries or by hormonally regulating cycles in models involving ovariectomy.

### Localizing Lesions

Locating endometriotic lesions in mice, particularly for longitudinal *in vivo* imaging designs, can present unique challenges. A model incorporating a luminescence strategy in donor endometrium is a key enabler in this context (Hirata et al., 2005). In particular, the use of fluorophores with high signal-to-noise characteristics allows small lesions, and lesions lacking a classical appearance, to be detected. Locating lesions with fluorescence is important to ensure capture of the full spectrum of lesion types and locations.

## Conclusion

**FIGURE 1:**
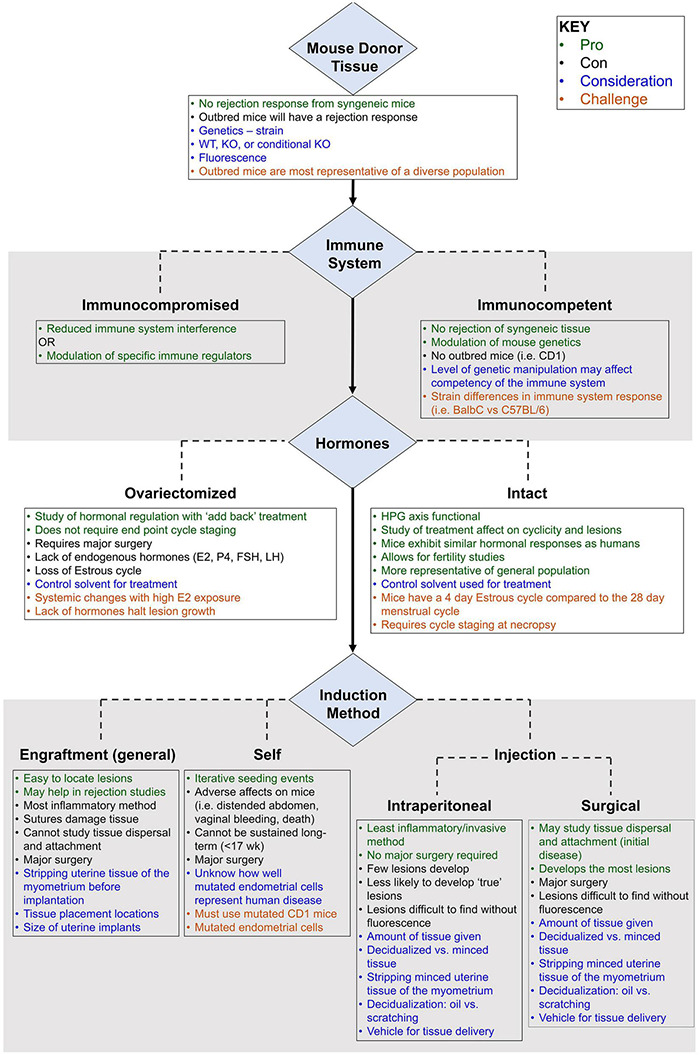
Primary considerations in the development of a mouse model of endometriosis using mouse donor tissue (homologous model). Variables (blue), challenges (burnt orange), pros (green), and cons (black) are highlighted.

**FIGURE 2:**
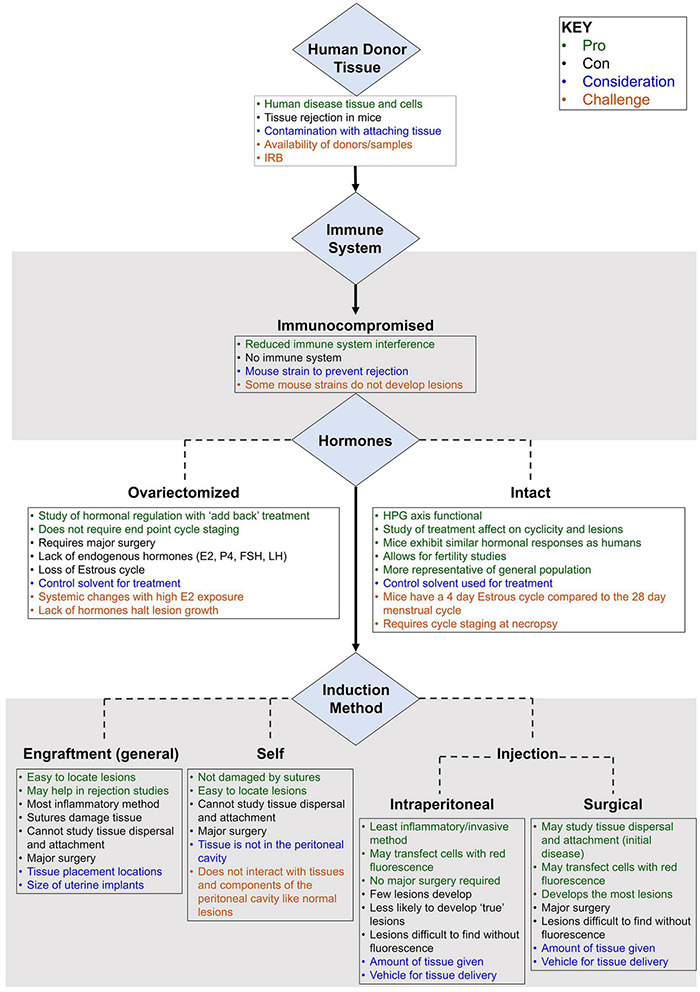
Primary considerations in the development of a mouse model of endometriosis using human donor tissue (heterologous model). Variables (blue), challenges (burnt orange), pros (green), and cons (black) are highlighted.

Preclinical modeling using a cost-efficient and molecularly well-annotated species is fundamental to studies of pathogenesis, biomarker development, and preventive and therapeutic discovery. This is particularly true for complex disease, such as endometriosis, for which a non-surgical method of diagnosis and surveillance to support longitudinal clinical study designs is not currently available. The successful translation of promising discoveries identified in a preclinical model to human use is largely contingent on model fidelity. The selection of the mouse system in modeling endometriosis requires careful consideration of genetic background, hormonal cyclicity, immunocompetency, lesion detection strategy, need for spontaneous endometrial attachment, and possibly iterative seeding (Figures 1, 2). We reviewed the published murine models in the context of ideal parameters founded on well-evidenced pathophysiologic features described in endometriosis. Collectively, these models have provided important insights and steady advancement toward recapitulating the molecular hallmarks of this disease. Though gaps remain, murine models represent a powerful resource for translational research in endometriosis.

## Author Contributions

KB, RB, and AP contributed to the design of the review manuscript. JS, EP, and AS developed the initial database of relevant manuscript. AP, RB, and KB wrote the first draft of the manuscript and the data tables. KB, RB, and AP wrote sections of the manuscript. NE conceptualized the figures. All the authors contributed to the manuscript revision, read, and approved the submitted version.

## Author Disclaimer

The views expressed are those of the authors and do not reflect the official policy or position of the Department of the Army, Department of Defense, or the United States Government.

## Conflict of Interest

The authors declare that the research was conducted in the absence of any commercial or financial relationships that could be construed as a potential conflict of interest.

## Publisher’s Note

All claims expressed in this article are solely those of the authors and do not necessarily represent those of their affiliated organizations, or those of the publisher, the editors and the reviewers. Any product that may be evaluated in this article, or claim that may be made by its manufacturer, is not guaranteed or endorsed by the publisher.

## References

[B1] Ajayi A. F. Akhigbe R. E. (2020). Staging of the estrous cycle and induction of estrus in experimental rodents: an update. *Fertil. Res. Pract.* 6:5. 10.1186/s40738-020-00074-3 32190339 PMC7071652

[B2] Alali Z. Graham A. Swan K. Flyckt R. Falcone T. Cui W. (2020). 60S acidic ribosomal protein P1 (RPLP1) is elevated in human endometriotic tissue and in a murine model of endometriosis and is essential for endometriotic epithelial cell survival in vitro. *Mol. Hum. Reprod.* 26 53–64. 10.1093/molehr/gaz065 31899515 PMC8204708

[B3] Alsina-Sanchis E. Mülfarth R. Moll I. Mogler C. Rodriguez-Vita J. Fischer A. (2021). Intraperitoneal oil application causes local inflammation with depletion of resident peritoneal macrophages. *Mol. Cancer Res.* 19 288–300. 10.1158/1541-7786.MCR-20-0650 33139505

[B4] Altan Z. M. Denis D. Kagan D. Grund E. M. Palmer S. S. Nataraja S. G. (2010). A long-acting tumor necrosis factor alpha-binding protein demonstrates activity in both in vitro and in vivo models of endometriosis. *J. Pharmacol. Exp. Ther.* 334 460–466. 10.1124/jpet.110.166488 20435921

[B5] Bacci M. Capobianco A. Monno A. Cottone L. Di Puppo F. Camisa B. (2009). Macrophages are alternatively activated in patients with endometriosis and required for growth and vascularization of lesions in a mouse model of disease. *Am. J. Pathol.* 175 547–556. 10.2353/ajpath.2009.081011 19574425 PMC2716955

[B6] Battersby S. Critchley H. O. de Brum-Fernandes A. J. Jabbour H. N. (2004). Temporal expression and signalling of prostacyclin receptor in the human endometrium across the menstrual cycle. *Reproduction* 127 79–86. 10.1530/rep.1.00038 15056772 PMC2694990

[B7] Becker C. M. Beaudry P. Funakoshi T. Benny O. Zaslavsky A. Zurakowski D. (2011). Circulating endothelial progenitor cells are up-regulated in a mouse model of endometriosis. *Am. J. Pathol.* 178 1782–1791. 10.1016/j.ajpath.2010.12.037 21435458 PMC3070089

[B8] Becker C. M. Rohwer N. Funakoshi T. Cramer T. Bernhardt W. Birsner A. (2008). 2-Methoxyestradiol inhibits hypoxia-inducible factor-1{alpha} and suppresses growth of lesions in a mouse model of endometriosis. *Am. J. Pathol.* 172 534–544. 10.2353/ajpath.2008.061244 18202195 PMC2312351

[B9] Becker C. M. Wright R. D. Satchi-Fainaro R. Funakoshi T. Folkman J. Kung A. L. (2006). A novel noninvasive model of endometriosis for monitoring the efficacy of antiangiogenic therapy. *Am. J. Pathol.* 168 2074–2084. 10.2353/ajpath.2006.051133 16723720 PMC1606629

[B10] Bellofiore N. Ellery S. J. Mamrot J. Walker D. W. Temple-Smith P. Dickinson H. (2017). First evidence of a menstruating rodent: the spiny mouse (Acomys cahirinus). *Am. J. Obstet. Gynecol*. 216 40.e1–40. e11. 10.1016/j.ajog.2016.07.041 27503621

[B11] Bilotas M. Meresman G. Stella I. Sueldo C. Baranao R. I. (2010). Effect of aromatase inhibitors on ectopic endometrial growth and peritoneal environment in a mouse model of endometriosis. *Fertil. Steril.* 93 2513–2518. 10.1016/j.fertnstert.2009.08.058 19819437

[B12] Bindels D. S. Haarbosch L. van Weeren L. Postma M. Wiese K. E. Mastop M. (2017). mScarlet: a bright monomeric red fluorescent protein for cellular imaging. *Nat. Methods* 14 53–56. 10.1038/nmeth.4074 27869816

[B13] Blumenkrantz M. J. Gallagher N. Bashore R. A. Tenckhoff H. (1981). Retrograde menstruation in women undergoing chronic peritoneal dialysis. *Obstet. Gynecol.* 57 667–670. 7219918

[B14] Buck Louis G. M. M. L. Hediger M. L. Peterson C. M. Croughan M. Sundaram R. Stanford J. (2011). Incidence of endometriosis by study population and diagnostic method: the ENDO study. *Fertil. Steril.* 96 360–365. 10.1016/j.fertnstert.2011.05.087 21719000 PMC3143230

[B15] Burney R. O. Giudice L. C. (2012). Pathogenesis and pathophysiology of endometriosis. *Fertil. Steril.* 98 511–519. 10.1016/j.fertnstert.2012.06.029 22819144 PMC3836682

[B16] Burns K. A. Rodriguez K. F. Hewitt S. C. Janardhan K. S. Young S. L. Korach K. S. (2012). Role of estrogen receptor signaling required for endometriosis-like lesion establishment in a mouse model. *Endocrinology* 153 3960–3971. 10.1210/en.2012-1294 22700766 PMC3404357

[B17] Burns K. A. Thomas S. Y. Hamilton K. J. Young S. L. Cook D. N. Korach K. S. (2018). Early endometriosis in females is directed by immune-mediated estrogen receptor alpha and IL-6 cross-talk. *Endocrinology* 159 103–118. 10.1210/en.2017-00562 28927243 PMC5761597

[B18] Cao X. Yang D. Song M. Murphy A. Parthasarathy S. (2004). The presence of endometrial cells in the peritoneal cavity enhances monocyte recruitment and induces inflammatory cytokines in mice: implications for endometriosis. *Fertil. Steril.* 82(Suppl. 3) 999–1007. 10.1016/j.fertnstert.2004.04.040 15474064

[B19] Chadchan S. B. Cheng M. Parnell L. A. Yin Y. Schriefer A. I. Mysorekar U. (2019). Antibiotic therapy with metronidazole reduces endometriosis disease progression in mice: a potential role for gut microbiota. *Hum. Reprod.* 34 1106–1116. 10.1093/humrep/dez041 31037294 PMC6554192

[B20] Chang L. C. Chiang Y. F. Chen H. Y. Huang Y. J. Liu A. C. Hsia S. M. (2020). The potential effect of fucoidan on inhibiting epithelial-to-mesenchymal transition, proliferation, and increase in apoptosis for endometriosis treatment: in vivo and in vitro study. *Biomedicines* 8:528. 10.3390/biomedicines8110528 33266505 PMC7700274

[B21] Chen P. Mamillapalli R. Habata S. Taylor H. S. (2021). Endometriosis stromal cells induce bone marrow mesenchymal stem cell differentiation and PD-1 expression through paracrine signaling. *Mol. Cell Biochem.* 476 1717–1727. 10.1007/s11010-020-04012-1 33428059

[B22] Chen Q. Zhou W. Pu D. Li Z. Huang Q. Chen Q. (2009). The inhibitory effect of 15-R-LXA4 on experimental endometriosis. *Eur. J. Obstet. Gynecol. Reprod. Biol.* 145 200–204. 10.1016/j.ejogrb.2009.05.015 19523744

[B23] Chen S. Bennet L. McGregor A. L. (2015). MacGreen mice: a novel tool to investigate inflammation following experimental stroke. *J. Exp. Stroke Trans. Med.* 8 1–9. 10.4172/1939-067X.1000143

[B24] Cheng C. W. Licence D. Cook E. Luo F. Arends M. J. Smith S. K. (2011). Activation of mutated K-ras in donor endometrial epithelium and stroma promotes lesion growth in an intact immunocompetent murine model of endometriosis. *J. Pathol.* 224 261–269. 10.1002/path.2852 21480232

[B25] Cohen J. Naoura I. Castela M. Von N’Guyen T. Oster M. Fontaine R. (2014). Pregnancy affects morphology of induced endometriotic lesions in a mouse model through alteration of proliferation and angiogenesis. *Eur. J. Obstet. Gynecol. Reprod. Biol.* 183 70–77. 10.1016/j.ejogrb.2014.10.038 25461356

[B26] Cohen J. Ziyyat A. Naoura I. Chabbert-Buffet N. Aractingi S. Darai E. (2015). Effect of induced peritoneal endometriosis on oocyte and embryo quality in a mouse model. *J. Assist. Reprod. Genet.* 32 263–270. 10.1007/s10815-014-0390-1 25399065 PMC4354196

[B27] Colón-Caraballo M. García M. Mendoza A. Flores I. (2019). Human endometriosis tissue microarray reveals site-specific expression of estrogen receptors. progesterone receptor, and Ki67. *Appl. Immunohistochem. Mol. Morphol.* 27 491–500. 10.1097/PAI.0000000000000663 29629944 PMC6174114

[B28] Cummings A. M. Metcalf J. L. (1995). Induction of endometriosis in mice: a new model sensitive to estrogen. *Reprod. Toxicol.* 9 233–238. 10.1016/0890-6238(95)00004-t 7579907

[B29] Cunha G. R. Sinclair A. Ricke W. A. Robboy S. J. Cao M. Baskin L. S. (2019). Reproductive tract biology: of mice and men. *Differentiation* 110 49–63. 10.1016/j.diff.2019.07.004 31622789 PMC7339118

[B30] Dabrosin C. Gyorffy S. Margetts P. Ross C. Gauldie J. (2002). Therapeutic effect of angiostatin gene transfer in a murine model of endometriosis. *Am. J. Pathol.* 161 909–918. 10.1016/S0002-9440(10)64251-4 12213719 PMC1867254

[B31] De Graaff A. A. DHooghe T. M. Dunselman G. A. Dirksen C. D. Hummelshoj L. Werf EndoCost Consortium (2013). The significant effect of endometriosis on physical, mental and social wellbeing: results from an international cross-sectional survey. *Hum. Reprod.* 28 2677–2685. 10.1093/humrep/det284 23847114

[B32] Defrere S. Colette S. Lousse J. C. Donnez J. Van Langendonckt A. (2009). Review: luminescence as a tool to assess pelvic endometriosis development in murine models. *Reprod. Sci.* 16 1117–1124. 10.1177/1933719109335069 19380902

[B33] D’Hooghe T. M. (1997). Clinical relevance of the baboon as a model for the study of endometriosis. *Fertil. Steril.* 68 613–625. 10.1016/s0015-0282(97)00277-x 9341599

[B34] D’Hooghe T. M. Bambra C. S. Jonge I. De Lauweryns J. M. Raeymaekers B. M. Koninckx P. R. (1997). The effect of pregnancy on endometriosis in baboons (*Papio anubis. Papio cynocephalus*). *Arch. Gynecol. Obstet.* 261 15–19. 10.1007/s004040050191 9451518

[B35] D’Hooghe T. M. Bambra C. S. Raeymaekers B. M. Koninckx P. R. (1996). Development of spontaneous endometriosis in baboons. *Obstet. Gynecol.* 88 462–466. 10.1016/0029-7844(96)00205-0 8752259

[B36] D’Hooghe T. M. Bambra C. S. Raeymaekers B. M. Jonge I. De Lauweryns J. M. Koninckx P. R. (1995). Intrapelvic injection of menstrual endometrium causes endometriosis in baboons (*Papio cynocephalus* and *Papio anubis*). *Am. J. Obstet. Gynecol.* 173 125–134. 10.1016/0002-9378(95)90180-9 7631669

[B37] D’Hooghe T. M. Bambra C. S. Suleman M. A. Dunselman G. A. Evers H. L. Koninckx P. R. (1994). Development of a model of retrograde menstruation in baboons (*Papio anubis*). *Fertil. Steril.* 62 635–638. 10.1016/s0015-0282(16)56957-x 8062962

[B38] Dlugi A. M. Miller J. D. Knittle J. (1990). Lupron**TAP Pharmaceuticals, North Chicago, Illinois. depot (leuprolide acetate for depot suspension) in the treatment of endometriosis: a randomized, placebo-controlled, double-blind study †† Supported by a grant from TAP Pharmaceuticals, North Chicago, Illinois. *Fertil. Steril.* 54 419–427. 10.1016/s0015-0282(16)53755-8 2118858

[B39] Dodds K. N. Beckett E. A. H. Evans S. F. Hutchinson M. R. (2017). Lesion development is modulated by the natural estrous cycle and mouse strain in a minimally invasive model of endometriosis. *Biol. Reprod.* 97 810–821. 10.1093/biolre/iox132 29069288

[B40] Dorning A. Dhami P. Panir K. Hogg C. Park E. Ferguson G. D. (2021). Bioluminescent imaging in induced mouse models of endometriosis reveals differences in four model variations. *Dis. Model. Mech.* 14:dmm049070. 10.1242/dmm.049070 34382636 PMC8419713

[B41] Edwards A. K. Nakamura D. S. Virani S. Wessels J. M. Tayade C. (2013). Animal models for anti-angiogenic therapy in endometriosis. *J. Reprod. Immunol.* 97 85–94. 10.1016/j.jri.2012.10.012 23432875

[B42] Efstathiou J. A. Sampson D. A. Levine Z. Rohan R. M. Zurakowski D. Folkman J. (2005). Nonsteroidal antiinflammatory drugs differentially suppress endometriosis in a murine model. *Fertil. Steril.* 83 171–181. 10.1016/j.fertnstert.2004.06.058 15652904

[B43] Eskenazi B. Warner M. L. (1997). Epidemiology of endometriosis. *Obstet. Gynecol. Clin. North Am.* 24 235–258. 10.1016/S0889-8545(05)70302-8 9163765

[B44] Fainaru O. Adini A. Benny O. Adini I. Short S. Bazinet L. (2008). Dendritic cells support angiogenesis and promote lesion growth in a murine model of endometriosis. *FASEB J.* 22 522–529. 10.1096/fj.07-9034com 17873101

[B45] Fattori V. Franklin N. S. Gonzalez-Cano R. Peterse D. Ghalali A. Madrian E. (2020). Nonsurgical mouse model of endometriosis-associated pain that responds to clinically active drugs. *Pain* 161 1321–1331. 10.1097/j.pain.0000000000001832 32132396

[B46] Ferrero H. Buigues A. Martinez J. Simon C. Pellicer A. Gomez R. (2017). A novel homologous model for noninvasive monitoring of endometriosis progression. *Biol. Reprod.* 96 302–312. 10.1095/biolreprod.116.140756 28203742

[B47] Fink D. Wohrer S. Pfeffer M. Tombe T. Ong C. J. Sorensen P. H. (2010). Ubiquitous expression of the monomeric red fluorescent protein mCherry in transgenic mice. *Genesis* 48 723–729. 10.1002/dvg.20677 20853428

[B48] Forster R. Sarginson A. Velichkova A. Hogg C. Dorning A. Horne A. W. (2019). Macrophage-derived insulin-like growth factor-1 is a key neurotrophic and nerve-sensitizing factor in pain associated with endometriosis. *FASEB J.* 33 11210–11222. 10.1096/fj.201900797R 31291762 PMC6766660

[B49] Giudice L. C. Kao L. C. (2004). Endometriosis. *Lancet* 364 1789–1799. 10.1016/S0140-6736(04)17403-5 15541453

[B50] Greaves E. Cousins F. L. Murray A. Esnal-Zufiaurre A. Fassbender A. Horne A. W. (2014). A novel mouse model of endometriosis mimics human phenotype and reveals insights into the inflammatory contribution of shed endometrium. *Am. J. Pathol.* 184 1930–1939. 10.1016/j.ajpath.2014.03.011 24910298 PMC4076466

[B51] Groothuis P. G. Dassen H. H. Romano A. Punyadeera C. (2007). Estrogen and the endometrium: lessons learned from gene expression profiling in rodents and human. *Hum. Reprod. Update* 13 405–417. 10.1093/humupd/dmm009 17584823

[B52] Halis G. Arici A. (2004). Endometriosis and inflammation in infertility. *Ann. N.Y. Acad Sci.* 1034 300–315. 10.1196/annals.1335.032 15731321

[B53] Halme J. Hammond M. G. Hulka J. F. Raj S. G. Talbert L. M. (1984). Retrograde menstruation in healthy women and in patients with endometriosis. *Obstet. Gynecol.* 64 151–154. 6234483

[B54] Hattori K. Ito Y. Honda M. Sekiguchi K. Hosono K. Shibuya M. (2020). Lymphangiogenesis induced by vascular endothelial growth factor receptor 1 signaling contributes to the progression of endometriosis in mice. *J. Pharmacol. Sci.* 143 255–263. 32487450 10.1016/j.jphs.2020.05.003

[B55] Hayashi S. Nakamura T. Motooka Y. Ito F. Jiang L. Akatsuka S. (2020). Novel ovarian endometriosis model causes infertility via iron-mediated oxidative stress in mice. *Redox Biol.* 37:101726. 10.1016/j.jphs.2020.05.003 PMC7509075 32961443

[B56] Heard M. E. Melnyk S. B. Simmen F. A. Yang Y. Pabona J. M. Simmen R. C. (2016). High-fat diet promotion of endometriosis in an immunocompetent mouse model is associated with altered peripheral and ectopic lesion redox and inflammatory status. *Endocrinology* 157 2870–2882. 10.1210/en.2016-1092 27175969 PMC4929556

[B57] Hirata T. Osuga Y. Yoshino O. Hirota Y. Harada M. Takemura Y. (2005). Development of an experimental model of endometriosis using mice that ubiquitously express green fluorescent protein. *Hum. Reprod.* 20 2092–2096. 10.1093/humrep/dei012 15831509

[B58] Horne A. W. Ahmad S. F. Carter R. Simitsidellis I. Greaves E. Hogg C. (2019). Repurposing dichloroacetate for the treatment of women with endometriosis. *Proc. Natl. Acad. Sci. U.S.A.* 116 25389–25391. 10.1073/pnas.1916144116 31792175 PMC6925989

[B59] Hsu A. L. Khachikyan I. Stratton P. (2010). Invasive and noninvasive methods for the diagnosis of endometriosis. *Clin. Obstet. Gynecol.* 53 413–419. 10.1097/GRF.0b013e3181db7ce8 20436318 PMC2880548

[B60] Itoh H. Sashihara T. Hosono A. Kaminogawa S. Uchida M. (2011). Interleukin-12 inhibits development of ectopic endometriotic tissues in peritoneal cavity via activation of NK cells in a murine endometriosis model. *Cytotechnology* 63 133–141. 10.1007/s10616-010-9321-x 21404062 PMC3080483

[B61] Jensen J. R. Witz C. A. Schenken R. S. Tekmal R. R. (2010). A potential role for colony-stimulating factor 1 in the genesis of the early endometriotic lesion. *Fertil. Steril.* 93 251–256. 10.1016/j.fertnstert.2008.09.050 18990370 PMC2812666

[B62] Jones R. L. Lang S. A. Kendziorski J. A. Greene A. D. Burns K. A. (2018). Use of a mouse model of experimentally induced endometriosis to evaluate and compare the effects of bisphenol a and bisphenol af exposure. *Environ. Health Perspect.* 126:127004. 10.1289/EHP3802 PMC6371646 30675821

[B63] Kim T. H. Yu Y. Luo L. Lydon J. P. Jeong J. W. Kim J. J. (2014). Activated AKT pathway promotes establishment of endometriosis. *Endocrinology* 155 1921–1930. 10.1210/en.2013-1951 24605828 PMC3990849

[B64] Kim Y. S. Kim Y. J. Kim M. J. Lee S. J. Kwon H. Lee J. H. (2020). Novel medicine for endometriosis and its therapeutic effect in a mouse model. *Biomedicines* 8:619. 10.3390/biomedicines8120619 PMC7766695 33339236

[B65] Körbel C. Menger M. D. Laschke M. W. (2010). Size and spatial orientation of uterine tissue transplants on the peritoneum crucially determine the growth and cyst formation of endometriosis-like lesions in mice. *Hum. Reprod.* 25 2551–2558. 10.1093/humrep/deq201 20693239

[B66] Kulak J. Jr. Fischer C. Komm B. Taylor H. S. (2011). Treatment with bazedoxifene, a selective estrogen receptor modulator, causes regression of endometriosis in a mouse model. *Endocrinology* 152 3226–3232. 10.1210/en.2010-1010 21586552 PMC3138238

[B67] Kumar R. Clerc A. C. Gori I. Russell R. Pellegrini C. Govender L. (2014). Lipoxin A(4) prevents the progression of de novo and established endometriosis in a mouse model by attenuating prostaglandin E(2) production and estrogen signaling. *PLoS One* 9:e89742. 10.1371/journal.pone.0089742 PMC3933674 24587003

[B68] Kusakabe K. T. Abe H. Kondo T. Kato K. Okada T. Otsuki Y. (2010). DNA microarray analysis in a mouse model for endometriosis and validation of candidate factors with human adenomyosis. *J. Reprod. Immunol.* 85 149–160. 10.1016/j.jri.2010.02.008 20452033

[B69] Lang T. J. (2004). Estrogen as an immunomodulator. *Clin. Immunol.* 113 224–230. 10.1016/j.clim.2004.05.011 15507385

[B70] Laschke M. W. Körbel C. Rudzitis-Auth J. Gashaw I. Reinhardt M. Hauff P. (2010). High-resolution ultrasound imaging: a novel technique for the noninvasive in vivo analysis of endometriotic lesion and cyst formation in small animal models. *Am. J. Pathol.* 176 585–593. 10.2353/ajpath.2010.090617 20042678 PMC2808067

[B71] Lebovic D. I. Mueller M. D. Taylor R. N. (2001). Immunobiology of endometriosis. *Fertil. Steril.* 75 1–10. 10.1016/S0015-0282(00)01630-7 11163805

[B72] Lee B. Du H. Taylor H. S. (2009). Experimental murine endometriosis induces DNA methylation and altered gene expression in eutopic endometrium. *Biol. Reprod.* 80 79–85. 10.1095/biolreprod.108.070391 18799756 PMC2804809

[B73] Li W. N. Hsiao K. Y. Wang C. A. Chang N. Hsu P. L. Sun C. H. (2020). Extracellular vesicle-associated VEGF-C promotes lymphangiogenesis and immune cells infiltration in endometriosis. *Proc. Natl. Acad. Sci. U.S.A.* 117 25859–25868. 10.1073/pnas.1920037117 33004630 PMC7568311

[B74] Li Y. Adur M. K. Kannan A. Davila J. Zhao Y. Nowak R. A. (2016). Progesterone alleviates endometriosis via inhibition of uterine cell proliferation, inflammation and angiogenesis in an immunocompetent mouse model. *PLoS One* 11:e0165347. 10.1371/journal.pone.0165347 PMC5077092 27776183

[B75] Liang Y. Wu J. Wang W. Xie H. Yao S. (2019). Pro-endometriotic niche in endometriosis. *Reprod. Biomed. Online* 38 549–559. 10.1016/j.rbmo.2018.12.025 30772194

[B76] Liao C. J. Li P. T. Lee Y. C. Li S. H. Chu S. T. (2014). Lipocalin 2 induces the epithelial-mesenchymal transition in stressed endometrial epithelial cells: possible correlation with endometriosis development in a mouse model. *Reproduction* 147 179–187. 10.1530/REP-13-0236 24194573

[B77] Lilius H. Hästbacka T. Isomaa B. (1996). A combination of fluorescent probes for evaluation of cytotoxicity and toxic mechanisms in isolated rainbow trout hepatocytes. *Toxicol. In Vitro* 10 341–348. 10.1016/0887-2333(96)00015-X 20650214

[B78] Lu Y. Nie J. Liu X. Zheng Y. Guo S. W. (2010). Trichostatin A, a histone deacetylase inhibitor, reduces lesion growth and hyperalgesia in experimentally induced endometriosis in mice. *Hum. Reprod.* 25 1014–1025. 10.1093/humrep/dep472 20118114

[B79] Machado D. E. Palumbo A. Santos Jr., J. M Mattos R. M. dos T. A. Santos S. H (2014). A GFP endometriosis model reveals important morphological characteristics of the angiogenic process that govern benign and malignant diseases. *Histol. Histopathol.* 29 903–912. 24385307 10.14670/HH-29.903

[B80] MacKenzie W. F. Casey H. W. (1975). Animal model of human disease. Endometriosis. Animal model: endometriosis in rhesus monkeys. *Am. J. Pathol.* 80 341–344. 1163633 PMC1912929

[B81] Madisen L. Zwingman T. A. Sunkin S. M. Oh S. W. Zariwala H. A. Gu H. (2010). A robust and high-throughput Cre reporting and characterization system for the whole mouse brain. *Nat. Neurosci.* 13 133–140. 10.1038/nn.2467 20023653 PMC2840225

[B82] Malvezzi H. Marengo E. B. Podgaec S. Piccinato C. A. (2020). Endometriosis: current challenges in modeling a multifactorial disease of unknown etiology. *J. Transl. Med.* 18:311. 10.1186/s12967-020-02471-0 PMC7425005 32787880

[B83] Martinez J. Bisbal V. Marin N. Cano A. Gómez R. (2019). Noninvasive monitoring of lesion size in a heterologous mouse model of endometriosis. *J. Vis. Exp.* e58358. 10.3791/58358 30882775

[B84] Matsuzaki S. Canis M. Darcha C. Dechelotte P. J. Pouly J. L. Mage G. (2008). Effects of a protein kinase C inhibitor on the initial development of ectopic implants in a syngeneic mouse model of endometriosis. *Fertil. Steril.* 89 206–211. 10.1016/j.fertnstert.2007.02.045 17481625

[B85] Mattos R. M. Machado D. E. Perini J. A. Alessandra-Perini J. Meireles da Costaa N. O. Wiecikowski A. F. D. R. O (2019). Galectin-3 plays an important role in endometriosis development and is a target to endometriosis treatment. *Mol. Cell Endocrinol.* 486 1–10. 10.1016/j.mce.2019.02.007 30753853

[B86] Maybin J. A. Hirani N. Brown P. Jabbour H. N. Critchley H. O. (2011). The regulation of vascular endothelial growth factor by hypoxia and prostaglandin F(2)alpha during human endometrial repair. *J. Clin. Endocrinol. Metab.* 96 2475–2483. 10.1210/jc.2010-2971 21677035 PMC3380090

[B87] Meuleman C. Vandenabeele B. Fieuws S. Spiessens C. Timmerman D. DHooghe T. (2009). High prevalence of endometriosis in infertile women with normal ovulation and normospermic partners. *Fertil. Steril.* 92 68–74. 10.1016/j.fertnstert.2008.04.056 18684448

[B88] Mishra A. Galvankar M. Vaidya S. Chaudhari U. Modi D. (2020). Mouse model for endometriosis is characterized by proliferation and inflammation but not epithelial-to-mesenchymal transition and fibrosis. *J. Biosci.* 45:105. 10.1007/s12038-020-00073-y 32975232

[B89] Nakamura Y. Ishii J. Kondo A. (2013). Bright fluorescence monitoring system utilizing *Zoanthus sp*. green fluorescent protein (ZsGreen) for human G-protein-coupled receptor signaling in microbial yeast cells. *PLoS One* 8:e82237. 10.1371/journal.pone.0082237 PMC3855394 24340008

[B90] Naqvi H. Sakr S. Presti T. Krikun G. Komm B. Taylor H. S. (2014). Treatment with bazedoxifene and conjugated estrogens results in regression of endometriosis in a murine model. *Biol. Reprod.* 90:121. 10.1095/biolreprod.113.114165 PMC4093999 24740602

[B91] Nezhat C. Nezhat F. Nezhat C. (2012). Endometriosis: ancient disease, ancient treatments. *Fertil. Steril.* 98 S1–S62. 10.1016/j.fertnstert.2012.08.001 23084567

[B92] Nowak N. M. Fischer O. M. Gust T. C. Fuhrmann U. Habenicht U. F. Schmidt A. (2008). Intraperitoneal inflammation decreases endometriosis in a mouse model. *Hum. Reprod.* 23 2466–2474. 10.1093/humrep/den189 18653673 PMC2569845

[B93] Ono Y. Yoshino O. Hiraoka T. Sato E. Furue A. Nawaz A. (2021). CD206+?macrophage is an accelerator of endometriotic-like lesion via promoting angiogenesis in the endometriosis mouse model. *Sci. Rep.* 11:853. 10.1038/s41598-020-79578-3 PMC7807007 33441630

[B94] Pelch K. E. Schroder A. L. Kimball P. A. Sharpe-Timms K. L. Davis J. W. Nagel S. C. (2010). Aberrant gene expression profile in a mouse model of endometriosis mirrors that observed in women. *Fertil. Steril.* 93, 1615–1627.e18. 10.1016/j.fertnstert.2009.03.086 19473656 PMC2904074

[B95] Peterse D. Binda M. M. Vanhie D. F. O. A. Fassbender A. Vriens J. DHooghe T. M. (2018). Of Mice and Women: a laparoscopic mouse model for endometriosis. *J. Minim. Invasive Gynecol.* 25 578–579. 10.1016/j.jmig.2017.10.008 29032250

[B96] Peyneau M. Kavian N. Chouzenoux S. Nicco C. Jeljeli M. Toullec L. (2019). Role of thyroid dysimmunity and thyroid hormones in endometriosis. *Proc. Natl. Acad. Sci. U.S.A.* 116 11894–11899. 10.1073/pnas.1820469116 31142643 PMC6575600

[B97] Piatkevich K. D. Verkhusha V. V. (2011). Guide to red fluorescent proteins and biosensors for flow cytometry. *Methods Cell Biol.* 102 431–461. 10.1016/B978-0-12-374912-3.00017-1 21704849 PMC3987785

[B98] Pierzchalski K. Taylor R. N. Nezhat C. Jones J. W. Napoli J. L. Yang G. (2014). Retinoic acid biosynthesis is impaired in human and murine endometriosis. *Biol. Reprod.* 91:84. 10.1095/biolreprod.114.119677 PMC4435029 25143356

[B99] Pittaluga E. Costa G. Krasnowska E. Brunelli R. Lundeberg T. Porpora M. G. (2010). More than antioxidant: N-acetyl-L-cysteine in a murine model of endometriosis. *Fertil. Steril.* 94 2905–2908. 10.1016/j.fertnstert.2010.06.038 20655527

[B100] Poulos C. Soliman A. M. Tekin S. Agarwal S. K. (2021). Patient preferences for elagolix and leuprolide for treating endometriosis-related pain in the United States. *Expert. Rev. Pharmacoecon. Outcomes Res.* 21 1091–1099. 10.1080/14737167.2021.1832468 33140977

[B101] Rendi M. H. Muehlenbachs A. Garcia R. L. Boyd K. L. (2012). “17 - Female Reproductive System,” in *Comparative Anatomy and Histology*, eds Treuting P. M. Dintzis S. M. (San Diego, CA: Academic Press).

[B102] Ricci A. G. Olivares C. N. Bilotas M. A. Meresman G. F. Baranao R. I. (2011). Effect of vascular endothelial growth factor inhibition on endometrial implant development in a murine model of endometriosis. *Reprod. Sci.* 18 614–622. 21266664 10.1177/1933719110395406

[B103] Rogers P. A. DHooghe T. M. Fazleabas A. Gargett C. E. Giudice L. C. Montgomery G. W. (2009). Priorities for endometriosis research: recommendations from an international consensus workshop. *Reprod. Sci.* 16 335–346. 19196878 10.1177/1933719108330568 PMC3682634

[B104] Ruiz A. Rockfield S. Taran N. Haller E. Engelman R. W. Flores I. (2016). Effect of hydroxychloroquine and characterization of autophagy in a mouse model of endometriosis. *Cell Death Dis.* 7:e2059. 10.1038/cddis.2015.361 PMC4816166 26775710

[B105] Salamonsen L. A. Lathbury L. J. (2000). Endometrial leukocytes and menstruation. *Hum. Reprod. Update* 6 16–27. 10711826 10.1093/humupd/6.1.16

[B106] Salamonsen L. A. Woolley D. E. (1999). Menstruation: induction by matrix metalloproteinases and inflammatory cells. *J. Reprod. Immunol.* 44 1–27. 10530758 10.1016/s0165-0378(99)00002-9

[B107] Sampson J. A. (1927). Metastatic or embolic endometriosis, due to the menstrual dissemination of endometrial tissue into the venous circulation. *Am. J. Pathol.* 3:43. PMC1931779 19969738

[B108] Sanchez A. M. Quattrone F. Pannese M. Ulisse A. Candiani M. Diaz-Alonso J. (2017). The cannabinoid receptor CB1 contributes to the development of ectopic lesions in a mouse model of endometriosis. *Hum. Reprod.* 32 175–184. 27821707 10.1093/humrep/dew281

[B109] Santorelli S. Fischer D. P. Harte M. K. Laru J. Marshall K. M. (2021). In vivo effects of AZD4547, a novel fibroblast growth factor receptor inhibitor, in a mouse model of endometriosis. *Pharmacol. Res. Perspect.* 9:e00759. 10.1002/prp2.759 PMC8019068 33811484

[B110] Sasmono R. T. Oceandy D. Pollard J. W. Tong W. Pavli P. Wainwright B. J. (2003). A macrophage colony-stimulating factor receptor-green fluorescent protein transgene is expressed throughout the mononuclear phagocyte system of the mouse. *Blood* 101 1155–1163. 12393599 10.1182/blood-2002-02-0569

[B111] Sato J. Nasu M. Tsuchitani M. (2016). Comparative histopathology of the estrous or menstrual cycle in laboratory animals. *J. Toxicol. Pathol.* 29 155–162. 27559240 10.1293/tox.2016-0021 PMC4963617

[B112] Schaefer B. C. Schaefer M. L. Kappler J. W. Marrack P. Kedl R. M. (2001). Observation of antigen-dependent CD8+ T-cell/dendritic cell interactions in vivo. *Cell Immunol.* 214 110–122. 12088410 10.1006/cimm.2001.1895

[B113] Seaman W. E. Gindhart T. D. (1979). Effect of estrogen on natural killer cells. *Arthritis Rheum.* 22 1234–1240. 389248 10.1002/art.1780221110

[B114] Sekiguchi K. Ito Y. Hattori K. Inoue T. Hosono K. Honda M. (2019). VEGF receptor 1-expressing macrophages recruited from bone marrow enhances angiogenesis in endometrial tissues. *Sci. Rep.* 9:7037. 10.1038/s41598-019-43185-8 PMC6504918 31065021

[B115] Sharma P. Lee J. L. Tsai E. M. Chang Y. Suen J. L. (2021). n-Butyl benzyl phthalate exposure promotes lesion survival in a murine endometriosis model. *Int. J. Environ. Res. Public Health* 18:3640. 10.3390/ijerph18073640 PMC8036315 33807420

[B116] Silveira C. G. Finas D. Hunold P. Koster F. Stroschein K. Canny G. O. (2013). L1 cell adhesion molecule as a potential therapeutic target in murine models of endometriosis using a monoclonal antibody approach. *PLoS One* 8:e82512. 10.1371/journal.pone.0082512 PMC3853202 24324802

[B117] Simoens S. Dunselman G. Dirksen C. Hummelshoj L. Bokor A. Brandes I. (2012). The burden of endometriosis: costs and quality of life of women with endometriosis and treated in referral centres. *Hum. Reprod.* 27 1292–1299. 22422778 10.1093/humrep/des073

[B118] Somigliana E. Vigano P. Rossi G. Carinelli S. Vignali M. Panina-Bordignon P. (1999). Endometrial ability to implant in ectopic sites can be prevented by interleukin-12 in a murine model of endometriosis. *Hum. Reprod.* 14 2944–2950. 10601076 10.1093/humrep/14.12.2944

[B119] Symons L. K. Miller J. E. Tyryshkin K. Monsanto S. P. Marks R. M. Lingegowda H. (2020). Neutrophil recruitment and function in endometriosis patients and a syngeneic murine model. *FASEB J.* 34 1558–1575. 31914688 10.1096/fj.201902272R

[B120] Takai E. Taniguchi F. Nakamura K. Uegaki T. Iwabe T. Harada T. (2013). Parthenolide reduces cell proliferation and prostaglandin E2 [corrected] in human endometriotic stromal cells and inhibits development of endometriosis in the murine model. *Fertil. Steril.* 100 1170–1178. 23876538 10.1016/j.fertnstert.2013.06.028

[B121] Tal A. Tal R. Shaikh S. Gidicsin S. Mamillapalli R. Taylor H. S. (2019). Characterization of cell fusion in an experimental mouse model of endometriosisdagger. *Biol. Reprod.* 100 390–397. 30304517 10.1093/biolre/ioy221 PMC7302516

[B122] Taylor H. S. Giudice L. C. Lessey B. A. Abrao M. S. Kotarski J. David F. (2017). Treatment of endometriosis-associated pain with Elagolix, an Oral GnRH antagonist. *N. Engl. J. Med.* 377 28–40. 28525302 10.1056/NEJMoa1700089

[B123] Tejada M. Á Santos-Llamas A. I. Fernández-Ramírez J. Tarín J. J. Cano A. Gómez R. (2021). A reassessment of the therapeutic potential of a dopamine receptor 2 agonist (D2-AG) in endometriosis by comparison against a standardized antiangiogenic treatment. *Biomedicines* 9:269. 10.3390/biomedicines9030269 PMC8001569 33800198

[B124] The Jackson Laboratory (2006). *The Importance of Genetic Background in Mouse-Based Biomedical Research.* Available online at: https://www.jax.org/news-and-insights/2006/june/the-importance-of-genetic-background-in-mouse-based-biomedical-research (accessed April 10, 2021)

[B125] Tomio K. Kawana K. Taguchi A. Isobe Y. Iwamoto R. Yamashita A. (2013). Omega-3 polyunsaturated Fatty acids suppress the cystic lesion formation of peritoneal endometriosis in transgenic mouse models. *PLoS One* 8:e73085. 10.1371/journal.pone.0073085 PMC3769312 24039864

[B126] Tuttle A. H. Philip V. M. Chesler E. J. Mogil J. S. (2018). Comparing phenotypic variation between inbred and outbred mice. *Nat. Methods* 15 994–996. 30504873 10.1038/s41592-018-0224-7 PMC6518396

[B127] Uegaki T. Taniguchi F. Nakamura K. Osaki M. Okada F. Yamamoto O. (2015). Inhibitor of apoptosis proteins (IAPs) may be effective therapeutic targets for treating endometriosis. *Hum. Reprod.* 30 149–158. 25376458 10.1093/humrep/deu288 PMC4262468

[B128] Umezawa M. Tanaka N. Tainaka H. Takeda K. Ihara T. Sugamata M. (2009). Microarray analysis provides insight into the early steps of pathophysiology of mouse endometriosis model induced by autotransplantation of endometrium. *Life Sci.* 84 832–837. 19345696 10.1016/j.lfs.2009.03.015

[B129] Vercellini P. Viganò P. Barbara G. Buggio L. Somigliana E. (2019). Elagolix for endometriosis: all that glitters is not gold. *Hum. Reprod.* 34 193–199. 30551159 10.1093/humrep/dey368

[B130] Wang C. C. Xu H. Man G. C. Zhang T. Chu K. O. Chu C. Y. (2013). Prodrug of green tea epigallocatechin-3-gallate (Pro-EGCG) as a potent anti-angiogenesis agent for endometriosis in mice. *Angiogenesis* 16 59–69. 22948799 10.1007/s10456-012-9299-4

[B131] Wang N. Hong S. Tan J. Ke P. Liang L. Fei H. (2014). A red fluorescent nude mouse model of human endometriosis: advantages of a non-invasive imaging method. *Eur. J. Obstet. Gynecol. Reprod. Biol.* 176 25–30. 24630298 10.1016/j.ejogrb.2014.02.012

[B132] Watanabe H. Numata K. Ito T. Takagi K. Matsukawa A. (2004). Innate immune response in Th1- and Th2-dominant mouse strains. *Shock* 22 460–466. 15489639 10.1097/01.shk.0000142249.08135.e9

[B133] Wieser F. Wu J. Shen Z. Taylor R. N. Sidell N. (2012). Retinoic acid suppresses growth of lesions, inhibits peritoneal cytokine secretion, and promotes macrophage differentiation in an immunocompetent mouse model of endometriosis. *Fertil. Steril.* 97 1430–1437. 22464761 10.1016/j.fertnstert.2012.03.004 PMC3367060

[B134] Wilkosz S. Pullen N. de-Giorgio-Miller A. Ireland G. Herrick S. (2011). Cellular exchange in an endometriosis-adhesion model using GFP transgenic mice. *Gynecol. Obstet. Invest.* 72 90–97. 21778678 10.1159/000325826

[B135] Wilson M. R. Holladay J. Chandler R. L. (2020). A mouse model of endometriosis mimicking the natural spread of invasive endometrium. *Hum. Reprod.* 35 58–69. 31886851 10.1093/humrep/dez253 PMC8205619

[B136] Wilson M. R. Reske J. J. Holladay J. Wilber G. E. Rhodes M. Koeman J. (2019). ARID1A and PI3-kinase pathway mutations in the endometrium drive epithelial transdifferentiation and collective invasion. *Nat. Commun.* 10:3554. 10.1038/s41467-019-11403-6 PMC6686004 31391455

[B137] Woo J. H. Choi Y. S. Choi J. H. (2020). Iron-storage protein ferritin is upregulated in endometriosis and iron overload contributes to a migratory phenotype. *Biomedicines* 8:454. 10.3390/biomedicines8110454 PMC7694081 33121166

[B138] Yan D. Liu X. Guo S. W. (2019). The establishment of a mouse model of deep endometriosis. *Hum. Reprod.* 34 235–247. 30561644 10.1093/humrep/dey361

[B139] Yoshiki A. Moriwaki K. (2006). Mouse phenome research: implications of genetic background. *ILAR J.* 47 94–102. 16547366 10.1093/ilar.47.2.94

[B140] Yoshino O. Ono Y. Honda M. Hattori K. Sato E. Hiraoka T. (2020). Relaxin-2 may suppress endometriosis by reducing fibrosis, scar formation, and inflammation. *Biomedicines* 8:467. 10.3390/biomedicines8110467 PMC7693148 33142814

[B141] Yoshino O. Osuga Y. Koga K. Hirota Y. Hirata T. Ruimeng X. (2006). FR 167653, a p38 mitogen-activated protein kinase inhibitor, suppresses the development of endometriosis in a murine model. *J. Reprod. Immunol.* 72 85–93. 16890996 10.1016/j.jri.2005.02.004

[B142] Yuan M. Li D. Zhang Z. Sun H. An M. Wang G. (2018). Endometriosis induces gut microbiota alterations in mice. *Hum. Reprod.* 33 607–616. 29462324 10.1093/humrep/dex372

[B143] Zhao Y. Li Q. Katzenellenbogen B. S. Lau L. F. Taylor R. N. I Bagchi C. (2014). Estrogen-induced CCN1 is critical for establishment of endometriosis-like lesions in mice. *Mol. Endocrinol.* 28 1934–1947. 25321413 10.1210/me.2014-1080 PMC4250364

